# Influence of Oxidation and Dialysis of Phlorotannins on Bioactivity and Composition of Ultrasound-Assisted Extracts from *Ascophyllum nodosum*

**DOI:** 10.3390/md20110706

**Published:** 2022-11-11

**Authors:** Mauro Gisbert, Jorge Sineiro, Ramón Moreira

**Affiliations:** Chemical Engineering Department, Universidade de Santiago de Compostela, Campus Vida, 15782 Santiago de Compostela, Spain

**Keywords:** phlorotannins, oxidation, molecular size, DPPH radical scavenging activity, FTIR, ^1^H-NMR, bioactivity, fragmentation, size distribution

## Abstract

The isolation and chemical characterization of phlorotannins has gained special attention in recent years due to their specific health-promoting benefits. Flow-cell ultrasound-assisted extraction (90 W/cm^2^ of sonication power, 2 min of retention time and 20 g solvent/g algae of liquid–solid ratio) was carried out by using double-distilled water (WE) and acetone:water mixture (AWE) as extraction solvents. The AWE showed a higher total polyphenols content (TPC), carbohydrates (CHOs) and antioxidant activities than WE. However, when the WE was purified by using Amberlite XAD16 column, the purified WE (PWE) showed similar a TPC, decreased CHOs and increased antioxidant activity compared to WE. The oxidation of the PWE extract was evaluated under natural, forced and severe oxidation condition for 120 h. Only severe oxidation conditions were able to significantly reduce TPC and antioxidant activities. PWE was dialyzed (20, 10, 3.5 and 2 kDa). The main bioactive fraction of phlorotannins was obtained from 10 to 20 kDa. CHOs were distributed in fractions below 20 kDa. MALDI-TOF analysis was performed for PWE, PD20 and PD2 extracts to analyze the degree of polymerization of phlorotannins, which ranged from 4 to 17 phloroglucinol units/molecule. Fragmentation patterns allowed the proximate identification of several phlorotannins in *Ascophyllum nodosum* extracts.

## 1. Introduction

Phlorotannins are bioactive compounds that exclusively come from brown seaweeds (Phaeophyta) such as *Ascophyllum nodosum* (*A. nodosum*), which shows famous antioxidant, anti-coagulant and antitumor features, among other bioactive features [1]. Thus, *A. nodosum* has been proposed by several researchers as a source of bioactive components for applications in medicine, pharmaceutics, cosmetics and the food industry [2].

Phlorotannins are part of cell membranes and walls, acting as shelters against environmental stress [1,3], and are produced by the polymerization of the phloroglucinol molecule in the polyketide pathway reaction [4]. Phlorotannins comprise molecules with a wide range of molecular size, ranging from 0.252 up to 650 kDa [5]. 

**Figure 1:**
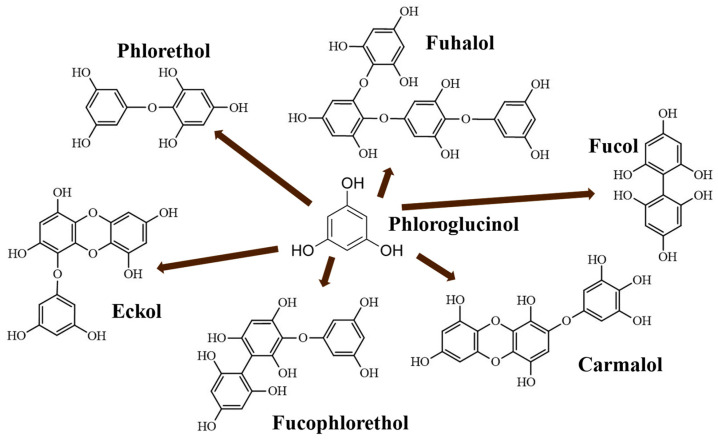
Phlorotannin main structures derived from phloroglucinol polyketide pathway reaction (adapted from Ford et al. [2]).

These molecules are classified into six different classes [2], attending to the variations of their assembling and distribution of hydroxyl groups: eckols, fuhalols, fucophlorethols, phlorethols, fucols and carmalols (Figure 1). Phlorethols and fuhalols present aryl-ether linkages; fucols aryl-aryl bonds; and fucophlorethols, a mixture of ether and phenyl bonds. Eckols present a 1,4-dibenzodioxin unit [6]. Carmalols present a 4-dibenzodioxin unit at the third and seventh position. Eckols differ from carmalols by their lower molecular weight and by the presence of an OH group substituted at the fourth carbon [7]. Fuhalols differ from phlorethols by their regular sequence of para- and ortho-ether bonds, by the presence of additional OH groups in every third ring and by the lack of one or more OH groups in the whole molecule [2].

Phlorotannins’ composition still remains scarcely studied, and very few commercial standards are available in the market [3]. Several isolation methods have been reported in the literature, but, in nature, these components are mainly high-molecular-weight polymers, and a depolymerization process is necessary [1,2,3,6].

Phlorotannins oxidation can be developed in seaweed tissues; this fact is suggested as one of the main steps in the complexation with carbohydrates or proteins during the formation of the cell membranes and walls [8,9]. Additionally, seaweeds’ processing (milling, drying, extraction and preservation) conditions can promote these reactions [10]. Xu et al. [11] suggested the degree of oxidation can be used as variable to control the bioactivity of phlorotannin itself, or to modify color or taste, as is performed in the production of tea, cacao or wine [12]. Quinones are the oxidized form of phlorotannins, and they partially maintain the bioactivity of original phloroglucinol derivatives; however, this fact is still unclear [13].

Ultrasound-assisted extraction (UAE) is highly effective to produce antioxidant-enriched extracts because it offers high operation reproducibility; offers reduced extraction time and solvent and energy consumption; allows for a continuous flow; and requires easy-to-manage and not-so-costly equipment [14,15]. Indeed, UAE is considered a novel and eco-friendly technique by the United States Environmental Protection Agency [16].

Phlorotannins’ extraction could be carried out with water, the safest solvent according to green chemistry principles, as a unique solvent [17]. Nonetheless, water shows lower extraction yields compared with organo-solvents, such as acetone or alcohols [4,17,18,19,20]. Moreover, water simultaneously solubilizes polysaccharides and proteins, producing extracts formed by a complex mixture of compounds [19].

It is not clear whether the extraction with organic solvents can promote the extraction of unbound phlorotannins [18] or, alternatively, if organo-solvents reduce the formation of phlorotannins complexes after they are extracted [4,21]. Thus, the selection of solvent type is critical and must be carried out with a higher polyphenol content, higher antioxidant activity and lower cost and time-consumption, together with the final use of phlorotannins [4,19].

The wide range of molecular sizes of phlorotannins (and/or complexation state) makes their separation, purification and characterization difficult due to the oligomeric structure and similar polarity of those biopolymers [5]. It was reported that the antioxidant capacities of phlorotannins are related to their structure and molecular size (degree of polymerization); in fact, oligomeric phlorotannins are generally more active than highly polymerized compounds [9].

The aim of the study was to generate phlorotannin-enriched extracts from *A. nodosum* by using methods based on both size (using different cutoff dialysis membranes) and oxidation state. Phlorotannins-enriched extracts were separated through dialysis membranes with different cutoff values, and the corresponding fractions were chemically analyzed. Additionally, the oxidation of phlorotannin-enriched fraction was studied to assess the resistance to oxidation of phlorotannins. Major components indicative of separation efficiency and bioactivity (total polyphenols content (TPC), total carbohydrates (CHOs) and DPPH radical scavenging activity (RSA)) were determined. Several spectrometry techniques (NMR, FTIR and MALDI–TOF) and a chromatography (RP-HPLC) analysis of the extracts were employed to support the identification of some phlorotannins in the different extracts and fractions.

## 2. Results and Discussion

### 2.1. Bioactivity Characterization

**Figure 2:**
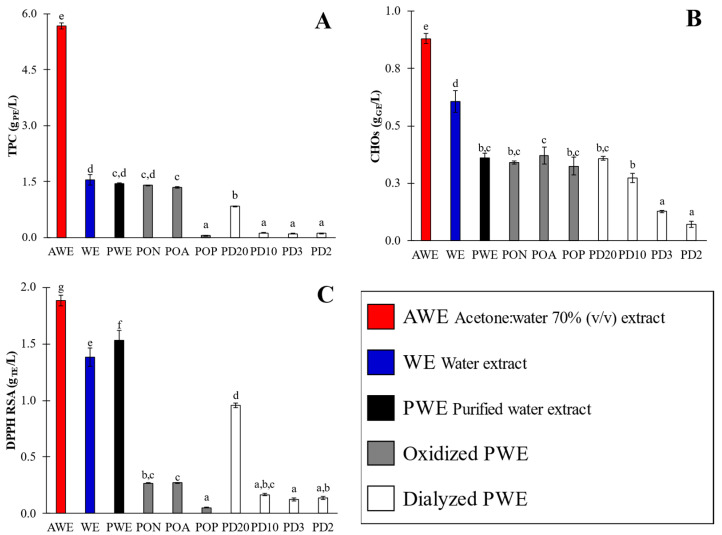
Phytochemical and antioxidant activity (TPC (**A**), CHOs (**B**) and DPPH RSA (**C**)) of crude (AWE and WE), purified (PWE), oxidized (PON, POA and POP) and dialyzed (PD20, PD10, PD3 and PD2) extracts. Different superscript letters indicate significant (*p* < 0.05) differences among values. Error bars correspond to standard deviations of measures.

Several phytochemical fractions were obtained from *A. nodosum*. Firstly, crude extracts were obtained from water (WE) and acetone:water (AWE) extraction; thereafter, the WE was purified by using an Amberlite XAD16 column to reduce the carbohydrates content (i.e., alginate isoforms) [10], and the purified WE fraction (PWE) was obtained. PWEs were subjected to several oxidation treatments, employing air (natural (PON) and forced aeration (POA)) and hydrogen peroxide (POP). Finally, PWEs were dialyzed by using 2–20 kDa cutoff cassettes that were given additional extracts (PD20, PD10, PD3 and PD2). The experimental values of the total polyphenol content (TPC), total carbohydrate content (CHOs) and antioxidant activity by DPPH scavenging method (RSA) of obtained extracts are shown in Figure 2.

The TPC values (Figure 2A) of crude extracts varied over a wide range, between 1.54 ± 0.14 and 5.67 ± 0.08 g PE/L; CHO values were in the range of 0.61 ± 0.05 to 0.88 ± 0.02 g GE/L; and RSA values were between 1.38 ± 0.08 and 1.88 ± 0.05 g TE/L. The highest content was obtained for AWE, evidencing the notorious effect of the extracting solvent type. Despite the fact that the AWE extract showed a higher TPC value, its antioxidant activity was not so notorious as could be expected (Figure 2C) when compared with that of WE. Hence, the AWE extract also showed a higher carbohydrate content than WE, evidencing that acetone:water 70% *v/v* is not so selective for polyphenols’ extraction as has been reported in several studies [4,18]. The AWE/WE calculated TPC ratio was 3.7 (Figure 2A), which was much higher than that obtained for CHOs (1.5, Figure 2B) and RSA values (1.4, Figure 2C). These results indicate that further separation of phlorotannins from carbohydrates (i.e., purification) is necessary to remove carbohydrates, mainly uronic acids in *A. nodosum*, when ultrasound-assisted extraction is performed, even when an acetone:water mixture is used as the solvent. 

From these results, WE was selected to be purified and to carry out further oxidation assays and fractioning by dialysis, because it is a reliable, cheap and food-friendlier solvent, as compared with acetone. PWE showed a similar TPC (TPC = 1.44 ± 0.02 g PE/L, PWE/WE = 1.1-fold higher) and RSA values (1.53 ± 0.09 g TE/L, 1.1-fold higher) to WE; however, with a significantly lower content of CHOs (0.36 ± 0.02 g GE/L, 1.8-fold lower). 

Hence, when PWE was compared with AWE, the ratios (±0.1) corresponding to TPC (TPC_AWE_/TPC_PWE_) and CHOs (CHOs_AWE_/CHOs_PWE_) were 3.9-fold and 2.4-fold lower, respectively, but RSA (RSA_AWE_/RSA_PWE_) was just 1.2-fold lower. These results evidenced that water permitted the extraction of phlorotannins with affinities for carbohydrates that significantly reduced the antioxidant activity of WE, as was also previously observed by Aleixandre et al. [10]; some authors referred to them as phlorotannin–carbohydrate complexes [19]. Thus, the purification of WE is highly recommended in order to achieve extracts that are comparable to AWE. Indeed, in previous studies, it was reported that Amberlite purification increased both the antioxidant and amylase- and glucosidase-enzyme-inhibiting activities of extracted polyphenols [10]. Thus, PWE extract, as an eco-friendlier option, was selected for further oxidation assays and dialysis to determine how bioactivity depends on the oxidation state and/or molecular size.

Catarino et al. [19] obtained *Fucus vesiculosus* extracts, and Wang et al. [22] obtained *A. nodosum* extracts, with TPC values of 0.86 and 0.28 g PE/L, respectively. Both authors studied phlorotannins’ extraction with 70% (*v*/*v*) acetone:water solution with conventional solid–liquid extraction, at a liquid–solid ratio of 20 g solvent/g algae for 24, at RT. The TPC values were significantly lower despite the very long conventional extraction (24 h) employed in comparison to values achieved in this study (5.67 ± 0.08 g PE/L) with UAE method. Zhang et al. [23] obtained similar TPC values of 5.6 g PE/L, applying conventional solid–liquid extraction, but employing 12.5 g solvent/g algae as the liquid–solid ratio, using water–ethanol (50% *v*/*v*) as the solvent and *A. nodosum* seaweed during long extraction times (90 min) and at a high temperature (80 °C). Ummat et al. [24] obtained extracts with TPC values of 4.8 g PE/L, using water–ethanol (50% *v*/*v*) as solvent, 10 g solvent/g algae as the liquid–solid ratio and long UAE times (30 min) from *Fucus vesiculosus* seaweed. Consequently, both authors reported close values to AWE extract (5.67 ± 0.08 g PE/L) but employing a higher temperature, longer time and lower liquid–solid ratio.

In the present research, limited oxidation of the PWE extract was performed under oxidation conditions varying from soft PON and POA to severe POP, with the aim of evaluating the extract’s remaining antioxidant activity in order to evaluate sensitivity of the phlorotannins-enriched extracts. The oxidation of PWE yielded outstanding results; The TPC values (Figure 2A) of oxidized extracts varied in a wide range, from a minimum value for POP (0.06 ± 0.001 g PE/L) to both PON and POA (1.4 ± 0.04). Regarding the PWE sample, natural and air-driven oxidation conditions caused minimal TPC reduction in both samples (1.6-fold lower), whereas POP showed a sharp TPC change (25-fold lower). Conversely, the CHOs’ results (Figure 2B) showed no significant (*p* < 0.05) differences among all oxidation assays (PON, POA and POP), with minor changes concerning the PWE extract (0.36 ± 0.02 g GE/L), with only 1.1-fold higher values, because the carbohydrates were not oxidable by hydrogen peroxide or oxygen in the assay conditions here applied. Nevertheless, the RSA values (Figure 2C) showed that all oxidation procedures had a notorious effect on antioxidant activity, for which the values varied from 0.05 ± 0.01 (POP) up to 0.27 ± 0.04 g TE/L (PON and POA), in contrast with an original value of PWE (1.53 g TE/L). Oxidation had a dramatic effect on antioxidant activity, reducing the RSA values by around 5.7-fold for PON and POA and around 30-fold for POP. The oxidation conditions indicate that polyphenols maintained their structure and content (TPC values), but their antioxidant capacities (RSA values) were reduced. Indeed, these results could also be used to assess the adequacy of the determination methods for screening antioxidants, whereas the TPC method could not show different values due to the existence of partially oxidized phlorotannins.

Dialysis of PWE extract was carried out throughout 20 (PD20), 10 (PD10), 3.5 (PD3) and 2 kDa (PD2) cutoff dialysis cassettes. The TPC values (Figure 2A) varied between 0.22 ± 0.01 (PD10, PD3 and PD2) and 0.84 ± 0.01 g PE/L (PD20). Similarly, RSA (Figure 2C) showed the highest values for PD20 (0.96 ± 0.02 g GE/L) and the other dialysates (0.14 ± 0.01). These results indicated that polyphenol molecules with sizes ranging from 2 up to 10 kDa were practically negligible (together with the obviously corresponding antioxidant activity). Finally, CHOs’ (Figure 2B) values increased with a dialysis cutoff size from 0.07 ± 0.01 (PD2) up to 0.36 ± 0.01 g GE/L (PD20). These results agree with those reported by Hermund et al. [25], who proposed that the relationship between bioactivity and molecular size is related to the enclosed structure of each phlorotannin and the amount unavailable OH-groups, both of which are dependent on the branching of the phlorotannins.

**Figure 3:**
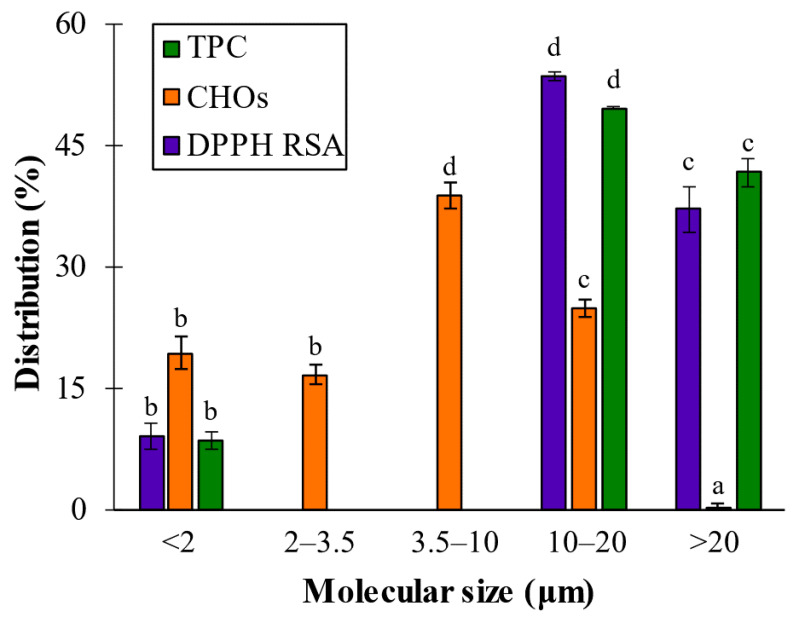
Distribution percentage of TPC, CHO and DPPH RSA values among dialyzed extracts. Different letters above bars mean that average values are significantly different (*p* > 0.95).

To compare the phytochemical composition data with other studies the percentage distribution of TPC, CHOs and RSA among the dialyzed fractions was calculated (Figure 3), assuming the total to be the content determined from PWE (TPC = 1.44 ± 0.020 g PE/L; RSA = 1.53 ± 0.09 and CHOs = 0.36 ± 0.02 g GE/L). CHOs were distributed between 0 and 20 kDa, in the following mode: <2 kDa fraction accounted for 19.4 ± 2.0% of CHOs; 2–3.5 kDa fraction rendered 16.7 ± 1.2%; 3.5–10 kDa fraction was the major fraction, accounting for 38.9 ± 1.5%; the 10–20 kDa fraction accounted for 25 ± 1.1%; and the negligible CHOs percentage was detected in the fraction with size higher than 20 kDa (0.2 ± 0.07%) (Figure 3).

In previous research in our laboratory, it was determined that the size of polymeric alginates extracted from *A. nodosum* after phlorotannins UAE ranged from 133 to 428 kDa [26]. However, no signals were obtained for sizes higher than 20 kDa, evidencing that purification and molecular size cutoff dialysis removed high-molecular-size alginic forms, and then CHOs’ signals corresponded to their smaller-sized oligomers that were found in all cutoff ranges.

The TPC and RSA analyses considering polyphenols’ molecular size distribution determined the existence of three main regions: lower than 2 kDa (8.5 ± 1.0 and 9.2 ± 1.7%), between 10 and 20 kDa (49.7 ± 0.2 and 53.6 ± 0.6%) and larger than 20 kDa (41.8 ± 1.7 and 37.3 ± 2.8%). These results showed that the largest population (around 50% of total TPC of the purified extract) and the most bioactive fraction (>53% of total RSA of the purified extract) was found at the 10–20 kDa range. These observations agreed with several authors who reported that the larger phlorotannins were the main bioactive compounds in the seaweeds, such as Audibert et al. [27], who found that polyphenol fractions >50 kDa showed the highest antioxidant activity, and Connan et al. [28], who reported that higher bioactivities were obtained when dialyzed extracts were around 100 kDa.

Nevertheless, both Tierney et al. [3] and Kubanek et al. [29] reported that phlorotannins from *A. nodosum* and *Fucus vesiculosus* were around 3.5–100 kDa (40–60% of polyphenols), and at least 32% of molecules were higher than 100 kDa. Paiva et al. [30], after investigating *Fucus spiralis* extracts, also reported that the highest bioactive fraction was obtained with 3 kDa ultra-filtrated extract. Bogolitsyn et al. [31] studied the molecular size of phlorotannins from *Fucus vesiculosus* seaweed, and it ranged from 8.7 to 49.3 kDa. They determined that the highest TPC value (0.98 g PE/g extract) was achieved with a fraction around 30 kDa, whereas the highest antioxidant activity (0.91 g AAE/g extract, expressed as g ascorbic acid equivalents per g extract) was obtained from the 14.5 kDa fraction, demonstrating that the fraction ranging from 8 to 18 kDa was the most active. The current study is agreement with these author’s findings, explaining the lower bioactivity of high-molecular-size phlorotannins due to conformational changes caused by the formation of intra- and inter-molecular bonds (i.e., bioaccessibility to phlorotannins is limited with their high molecular fractions). Then, to achieve the isolation and/or identification of the main phlorotannins group that provided the best results, additional characterization techniques were applied.

### 2.2. Chromatographic Characterization

**Figure 4:**
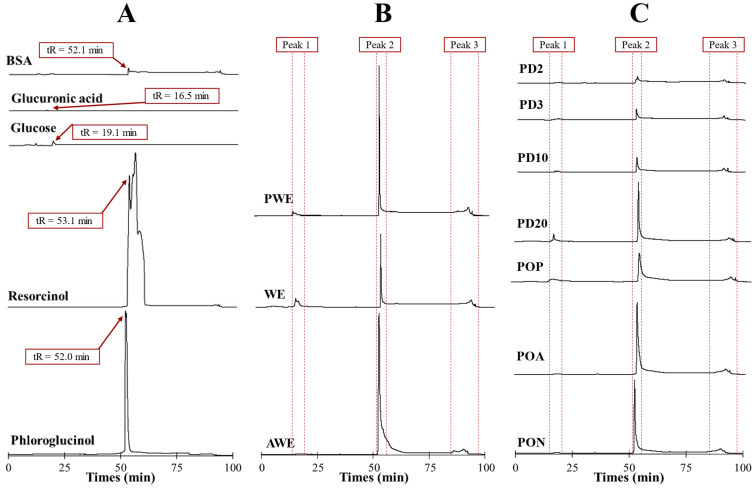
RP-HPLC–UV profiles (266 nm) of (**A**) standard compounds (phloroglucinol, resorcinol, glucose, glucuronic acid and BSA); (**B**) crude and purified (AWE, WE and PWE), (**C**) oxidized, (PON, POA and POP) and dialyzed (PD20, PD10, PD3 and PD2) extracts.

Nowadays there are no commercially available standards for phlorotannin polymers; resorcinol and phloroglucinol are usually employed [8]. Representative major compounds found in seaweeds were injected to test the elution pattern and deduce where oligomers of sugars, uronic acids, proteins and phlorotannin could be found. Additionally, the extracts’ profiles were compared with commercial D-(+)-glucose, D-(+)-glucuronic acid, bovine serum albumin (BSA), resorcinol and phloroglucinol to determine sugar/protein presence. The retention times (tR) of assayed standard compounds were 16.5, 19.1, 52.0, 52.1 and 52.1 min for glucuronic acid, glucose, phloroglucinol, BSA and resorcinol, respectively (Figure 4A). Detection was carried out at 266 nm and reported as maximal phloroglucinol absorption light [18]. Non-phenolic standards produced a much more minor signal compared with the prominent peaks observed from resorcinol and phloroglucinol (Figure 4A, Figure S1 for more detailed information at full scale), thus confirming that the main signals obtained from RP-HPLC could be assumed to be phlorotannins isomers.

The chromatograms of the extracts (Figure 4B,C) showed three clearly defined regions, as is common in all extracts, meaning that three main groups of molecules (i.e., same retention time) were present in all extracts with different content values (i.e., different peak intensity). The variation of signal intensities evidenced that the use of different solvents and subsequent post-treatments modified the phlorotannin profiles.

Peak 1 (tR = 16 ± 3 min) corresponded to the set of polar components that gave some absorbance at 266 nm and matched with glucose and glucuronic acid tR values. This peak was associated with the presence of oligosaccharides or proteins with low affinity that elute rapidly. Peak 2 (tR = 53 ± 2 min) corresponded to main phlorotannin isomers with relative similar affinity compound for C18 column that coincided with tR values of phloroglucinol and resorcinol standard injections. Additionally, Peak 2 was in all samples; it is the most notorious peak in comparison to Peaks 1 and 3. Finally, Peak 3 (tR = 91 ± 6 min) was associated with a high affinity for the column, assumed as high-molecular-size oligomers that eluted in a hump [18].

AWE showed a more intense Peak 2 signal compared with WE that, together with TPC values (Figure 2A), corroborated that acetone:water was a more efficient solvent for polyphenols’ extraction than water; however, PWE showed a prominent Peak 2 signal like AWE did. The differences between AWE and WE were related to their different content of phlorotannin–carbohydrate complexes. Tierney et al. [3] also reported no significant difference between water and the ethanol–water mixture, thus evidencing that the phlorotannins’ profile was not much different. Extraction with water or acetone:water produced co-extracted carbohydrates (Figure 2B); however, Peak 1 (Figure 4B) signals were hindered by the prominent signal of Peak 2. CHOs’ reduction after purification increased the amount of phlorotannin capable of interacting with the column (Peak 2) provided by the PWE sample. 

All oxidized PWEs (PON, POA and POP) showed a Peak 2 that was present in all samples, but its signal intensity was notoriously reduced because of the partial loss of OH groups (Figure 4C). Peak 3 signals did not show significant changes among oxidized samples. It was Peak 2 that showed the main difference among extracts (PON > POA > POP) after oxidation, whichreduced the signals of each treatment. The RP-HPLC results (Figure 3C) allowed us to differentiate between both soft oxidation conditions, where POA showed a major reduction in Peak 2 than just natural oxidation. Finally, and according to phytochemical characterizations (Figure 2), the POP extract showed a lower intensity peak concerning the PON, POA and PWE extracts. This effect evidenced that the bioactivity, together with the chemical nature of the POP sample, was altered after this more severe oxidation treatment.

All dialyzed PWE fractions (PD20 > PD10 > PD3 > PD2) showed lower peak signals than the original PWE (Figure 4C). Lower Peak 2 intensity of PD20 corroborated the proposed distribution of TPC and RSA values (49.7% and 53.6%), where the main bioactive fraction was found in the range from 10 to 20 kDa. Peak 1 signals were related to different small alginate oligomers that were still present after both treatments (dialysis and oxidation) of PWE, and their presence was shown in Figure 4C.

### 2.3. Spectroscopic Characterization

#### 2.3.1. FTIR

**Figure 5:**
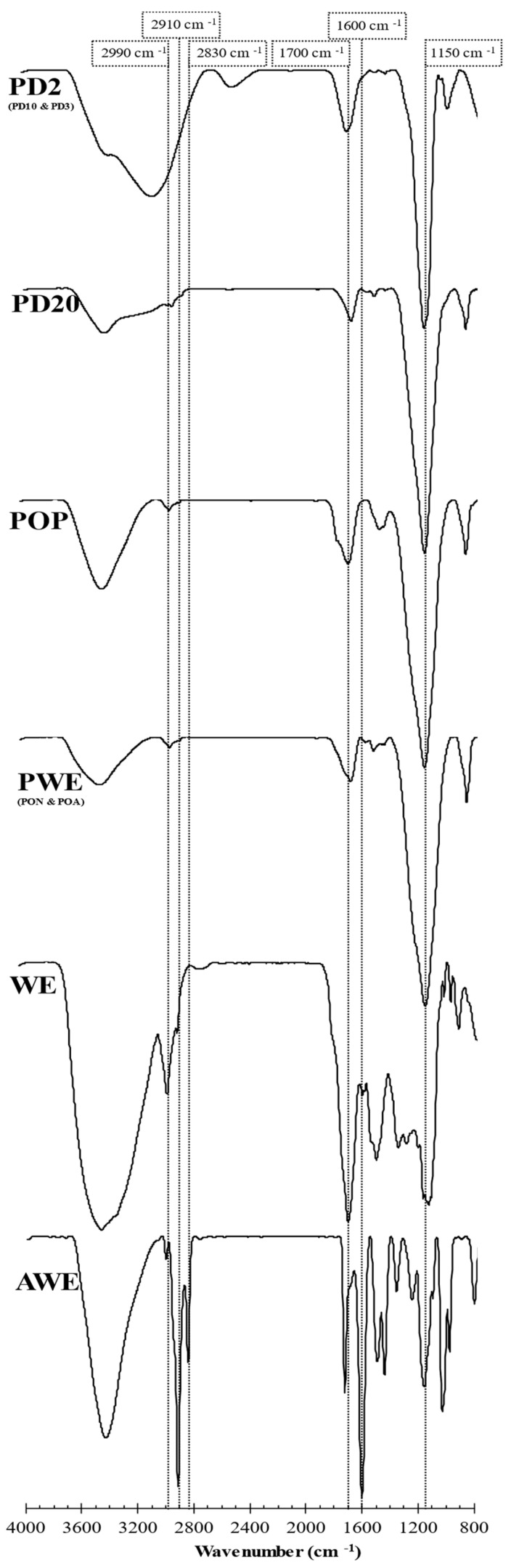
FTIR spectra of crude (AWE and WE), purified (PWE), oxidized (POP) and dialyzed (PD20 and PD2) extracts.

The FTIR technique was used to appreciate the notorious differences among assayed extracts, such as the number of peaks (due to chemical reactions), peak intensities (differences in compounds content) and peak shapes (presence of similar groups). The FTIR spectra for crude (AWE and WE), purified (PWE), oxidized (POP) and dialyzed (PD20 and PD2) extracts are shown in Figure 5. Oxidized POA and PON did not show significant differences with PWE, and dialyzed samples PD10 and PD3 showed similar spectra to PD2.

Three regions were evaluated as the main characteristic signals from polyphenolic compounds: Region 1 located around 2900 cm^−1^, Region 2 around 1600 cm^−1^ and Region 3 around 1100 cm^−1^. Region 1 was related to the vibrational stretch of inter- and intra-molecular hydroxyl groups. Hence, these peaks are often used as criterion to measure the strength of the hydrogen bonding of polar molecules [32]. Region 2 corresponded to phlorotannins and alginate signals. The 1700 and 1600 cm^−1^ are signals associated with aryl-aryl and ethyl ether bonds, both formed during the polymerization of phloroglucinol in the formation of phlorotannins. Thus, FTIR did not permit us to discern among phlorotannin families [33].

The FTIR spectra from two crude extracts (WE and AWE) showed several peaks in AWE slightly observed in WE (Figure 5). When Region 1 was analyzed, the spectrum of AWE showed three peaks, namely at 2830, 2910 and 3000 cm^−1^, whereas only the last two peaks were observed in the spectrum of WE. The peak around 3000 cm^−1^ showed a lower intensity in WE compared to AWE; this change was associated with the hydrogen bonding between phlorotannins and water, forming adducts that could explain the high intensity in WE rather than AWE. The prominent peak around 2830 cm^−1^ observed in AWE that was negligible in all the other samples was associated with traces of acetone. The signal around 2910 cm^−1^ could be explained as hydrogen bonding between phlorotannins and carbohydrates (and/or alginates).

AWE showed a relevant CHOs value, as WE did (Figure 2B), due to the lack of selectivity of UAE [17]. However, the 2910 cm^−1^ signal was dramatically reduced in PWE because of the removal of carbohydrates and alginate isoforms after purification treatment [10]. This fact was confirmed in oxidized and dialyzed extracts because this signal was minimally detected (Figure 5). The three signals in Region 1 were scarcely observed in the dialyzed samples. PD20 maintained the peak corresponding to water content at 3400 cm^−1^; however, PD2 showed a displaced signal that hindered hydrogen bonds’ signals in Region 1. This result was explained by the high accessibility of small phlorotannins (PD2) that can interact with solvent traces and sugars.

Region 2 is the characteristic zone of stretching vibrations of C=C bonds of aromatic rings, with signals of carboxylic acids, aldehydes and/or ketones [33]. Indeed, a 1600 cm^−1^ signal is associated with the asymmetric stretching vibrations of carboxylate vibration in alginic acid [26]. Then these signals at 1700 and 1600 cm^−1^ were associated with the presence of phlorotannins and alginate oligomers in the extracts. The crude extracts’ (AWE and WE) analysis showed similar intensities in phlorotannin content, thus differing from the TPC results (Figure 2A). A signal at 1700 cm^−1^ corresponded to different oxidation states that could be present in the enriched phlorotannin extracts. The PWE and PD20 samples showed similar intensity for the signal at 1700 cm^−1^ that increased in POP and PD2 samples. Similar FTIR spectra of POP and PD2, together with results of TPC (Figure 2A), RSA (Figure 2C) and Peak 2 of chromatograms (Figure 4B,C), suggested that the 1700 cm^−1^ signal was due to higher content in oxidized phlorotannin forms. 

A low content of alginates after purification was confirmed since their characteristic signal (1600 cm^−1^) was negligible in PWE extract and subsequent samples (oxidized and dialyzed). FTIR confirmed that purification treatment was suitable to remove alginates and other carbohydrates. Consequently, phlorotannins were more accessible, and, at the same TPC, the bioactivity increased (RSA value). Furthermore, it was previously reported that purified and crude extracts showed different anti-enzymatic activities [10]. The FTIR results were complemented with ^1^H-NMR and MALDI–TOF techniques to isolate possible chemical structures that explained those bioactivities that were observed in the extracts. 

#### 2.3.2. ^1^H-NMR

**Figure 6:**
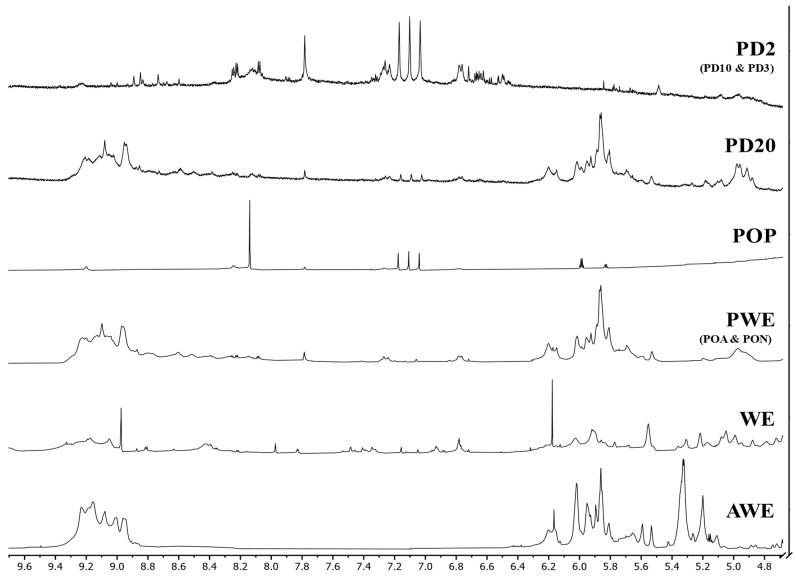
^1^H-NMR (DMSO-d_6_) spectra of crude (AWE and WE), purified (PWE), oxidized (POP) and dialyzed (PD20 and PD2) extracts.

An ^1^H-NMR analysis was carried out to give a proximate insight into the chemical structure of the crude (AWE and WE), purified (PWE), oxidized (POP) and dialyzed (PD20 and PD2) extracts. No significant differences were observed between POA and PON, with PWE and between PD10 and PD3 with PD2, similar to the results of FTIR spectroscopy. The ^1^H-NMR spectra of these samples showed noticeable signals in the range of 4.8–9.6 ppm (Figure 6), where the main polyphenolic compounds could be identified together [5,9,34,35].

The ^1^H-NMR of crude extracts (AWE and WE) showed noticeable differences (Figure 6). AWE showed two clearly differentiated areas where all the signals were aggregated, between 8.8 and 9.4 ppm and between 5.0 and 6.4 ppm, whereas the WE spectrum showed peaks throughout all the spectrum, with two noticeable peaks at 6.15 and 8.95 ppm, matching those groups of signals found in the AWE spectrum.

The observed differences between crude extracts could be related to the presence of carbohydrate–phlorotannin complexes. It seems that the acetone:water extraction minimized the binding of phlorotannins with other compounds, showing two main groups: one composed of almost pure phlorotannins, assigned to the high chemical shift (around 9 ppm), and a second group of partially bound (6 ppm) phlorotannins, closer to “sugars” region (<5 ppm). However, the use of water as a solvent seemed to promote complexation mechanisms, with several peaks appearing along the spectra and a wide variety of compounds (and/or complexes). This hypothesis was supported when PWE was analyzed (Figure 6) and it showed more similarities with AWE rather than WE.

Interestingly, it was found that oxidized PWE (PON, POA and POP) showed that ^1^H-NMR spectra of PON and POA (data not shown) did not show remarkable differences as compared with PWE samples. Hence, as was seen in the TPC (Figure 2A) and FTIR (Figure 5) results, soft oxidation conditions were not enough to alter proton profile shifts. Nevertheless, hydrogen peroxide oxidation dramatically changed the NMR spectrum. Three main groups of signals were observed in the POP spectrum: a peak around 8.2 ppm, three small peaks at 7.1 ppm, and two smaller peaks close to 5.9 ppm. Hydrogen peroxide oxidation was strong enough to almost remove the bioactive capacities of the extracts; however, the low signals explained their last remaining bioactive capacities (RSA, Figure 2C). Indeed, these signals could be further used as partial reference of quinones’ presence assessment.

Breton et al. [9] defined the purity of their samples by the apparition of exclusive peaks between 5.5 and 6.5 ppm. The authors did not report signals at chemical shifts higher than 8.8 (due to alcohols proton) because they were only visible with deuterated DMSO solvent. Thus, phlorotannin signals in AWE and PWE that seemed more abundant at a 5.0 to 6.4 ppm range were assumed to be an indicator of purity. When PWE dialysates were analyzed (Figure 6), a difference between PD20 and the other dialysates below 10 kDa (PD10, PD3 and PD2) was noted. PD20 showed some minor differences at 7.1 ppm, with three small peaks, similar to POP extract, that did not appear in PWE. PD20 after 120 h of dialysis treatment could contain quinones formed as self-oxidation process corresponding to that three peaks signal. According to the TPC (Figure 2A) and RSA (Figure 2C) values, dialysates below 10 kDa showed notorious differences as compared with the original PWE, as seen with FTIR (Figure 5). ^1^H-NMR of dialysated PWE showed peaks located between 6.4 and 8.4 ppm, in coincidence with three characteristic peaks found in the POP spectrum (around 7.0 ppm) corresponding to the oxidized polyphenols (quinones).

^1^H-NMR spectra similarities between the PWE and PD20 samples evidenced that the main bioactive fraction was obtained between 10 and 20 kDa, as it was observed previously in the analysis of components’ distribution (Figure 3). Additionally, the spectra were able to match the different bioactive capacities observed when comparing <2 kDa dialyzed samples with PD20 and PWE. Hence, this finding corroborated the low phlorotannins content in the dialysate fractions (<10 kDa) and that molecules were partially oxidized, explaining their low bioactivities, together with the Peak 2 signals observed in chromatographic profiles.

#### 2.3.3. MALDI–TOF-MS

The MALDI–TOF-MS analysis was performed to tentatively identify some of the phlorotannins extracted from *A. nodosum* seaweed, on of PWE and dialyzed PWE (PD20 and PD2), because these were the most pure and clean samples. The spectra of PWE ranged from 969 to 30,722 m/z, showing the complexity of PWE, whereas the major signals were found below 4000 m/z. PD20 ranged from 1001 up to 20,506 m/z, with wide separated peaks (1010–2797 m/z) by 162 m/z units. However, the PD20 sample still showed several hampered signals that made a suitable phlorotannin identification difficult. Finally, the PD2 extract ranged from 500 up to 1988 m/z and also showed separated peaks by 162 m/z (1988–529 m/z) and three peaks (586, 702 and 928 m/z) that did not match with the proposed fragmentation pattern. The mass fragment with m/z = 162 was observed by several authors, indicating that it can be related to the cross-ring cleavages [18].

**Figure 7:**
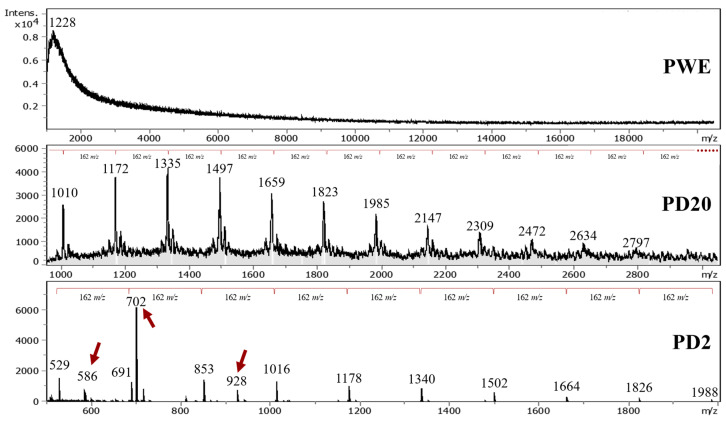
MALDI–TOF-MS spectra of purified (PWE) and dialyzed extracts (PD20 and PD2).

The identifications and degree of polymerization were determined based on the loss of phloroglucinol (PGU) moieties (126/125/124 m/z) and two hydroxyl/water (OH, H_2_O) moieties (17/18 m/z) that corresponded to 162 m/z separation of PD20 and PD2 samples (Figure 7). Phloroglucinol as the unique monomer of phlorotannins and the loss of water molecules (and/or OH groups) and deprotonation are effects generally considered during phlorotannins identification [8,18,20,34]. Thus, 1 PGU with 2 H_2_O molecules was used to determine the degree of polymerization (DP) of main signals, as shown in Equation (1):

(1) $$\mathrm{DP}\;=\frac{{\left[m/z\right]}_{m}+n\left[H_{2}O\right]}{\left[\mathrm{PHL}\right]+2\left[H_{2}O\right]}$$

where DP is the degree of polymerization in phloroglucinol units (PGU), [m/z]_m_ is the measured mass-to-charge ratio [m/z] of each peak, [PHL] is the [m/z] value of phloroglucinol, n is the number of water molecules lost and [H_2_O] is the [m/z] value of water.

The DP of dialyzed extracts showed that the phlorotannins that were extracted from *A. nodosum* seaweed by using UAE ranged from 4 to 9 and 7 to 17 PGUs for PD2 and PD20, respectively. Several reports from the literature confirmed that the average phlorotannin DP value ranges from around 3 up to 15 PGUs (Table 1); that could reach values of 39 PGUs [8].

Phlorotannins’ empirical formula was employed (Table 2) due to the several possible conformations of phloroglucinol that could be found in seaweed that are still under discussion [2,8]. The highest signal detected was around 702 m/z, as was also reported by Allwood et al. [20], without identification; it is assumed to be a fragmentation residue, and it is hypothesized that it could correspond to an isomer of the dimeric form of hydroxytrifuhalol A, as reported by Corona et al. [39], here forming an adduct with OH^−^ (811 − 126 + 17 = 702), or to a fragment of dieckol (C_30_O_22_H_18_) without two water molecules.

Allwood et al. [10] suggested that phlorotannins in *A. nodosum* seaweed were predominantly fucophlorethol-A type due to preponderance of cleavages of ether bonds, in agreement with Nwosu et al. [40], who reported that phlorotannins isolated from *A. nodosum* were derived from 7-phloroeckol. This result agrees with a previous FTIR analysis wherein an ethyl-ether signal (1410 cm^−1^) was prominent, especially for those derived from PWE (i.e., dialyzed and oxidized samples). Indeed, the obtained families in Table 2 were possibly related to the different group of phlorotannins (fucols, fuhalols, phlorethols, carmalols, eckols and fucophlorethols) shown in Figure 1. The main drawback of phlorotannins is that, despite the fact that they could have similar DP values or molecular sizes, they produce different fragmentation patterns [18] that do not allow for their identification.

Allwood et al. [10] found that dialysis could also modify the fragmentation patterns. Thus, another explanation for the lack of matching between obtained values in the present research and the literature data can be the consideration of the presence of oxidized molecules in PD2. The oxidation state showed their prominent effect in bioactive (Figure 2), chromatographic (Figure 4) and spectroscopic (FTIR in Figure 5, and ^1^H-NMR in Figure 6) characterizations, that could also affect the MALDI–TOF-MS analysis, producing a different fragmentation pattern. 

The obtained results contribute to the current literature for the fragmentation pattern and DP values of phlorotannins from *A. nodosum*. Moreover, the results derived from the present research may be further used to determine the potential presence of oxidized species of phlorotannins, thus possibly leading to a greater understanding of the nature and behavior of these complex and bioactive compounds.

## 3. Materials and Methods

### 3.1. Chemicals

All reagents used for characterization were analytical grade. We obtained 2,2-diphenyl-1-picrylhydrazyl (DPPH), phenol, DMSO-d_6_, H_2_O_2_ 36% (*v*/*v*), D-(+)-glucose, formic acid, sulfuric acid, resorcinol, glucose and glucuronic acid and phloroglucinol from Sigma-Aldrich (St. Louis, MO, USA). Sodium carbonate, albumin bovine serum (BSA), HPLC water, Folin–Ciocâlteu reagent, Amberlite XAD16 resin, Trolox and methanol we obtained from Panreac (Barcelona, Spain).

### 3.2. Raw Materials

Fresh *Ascophyllum nodosum* seaweed (*A. nodosum*) from the Galician Coasts (NW of Spain), harvested in November of 2019, was supplied by Mar de Ardora S.L. company (A Coruña, Spain); it was dried in a hot-air convective dryer Angelantoni Challenge 250, (Angelantonie Industrie S.p.A., Cimacole, Perugia, Italy) at 50 °C, at constant 30% relative humidity, load density of 2 kg/m^2^ and air velocity at 2 m/s until moisture content was 10.0 ± 0.8% (dry basis, d.b.). Dried samples were milled (276 ± 8 μm of mean particle size) in an ultra-centrifugal mill (Retsch GmbH, ZM200, Haan, Germany) and stored at 4 °C until their use.

### 3.3. Crude Extraction

Crude water and acetone extracts (WE and AWE) from *A. nodosum* were obtained by ultrasound-assisted extraction (UAE) with double-distilled water or a 70% acetone:water mixture. *A. nodosum* powder (1:20, 1 g algae powder per 20 g solvent) was soaked in the corresponding solvent, while stirring (175 rpm), for 15 min, at room temperature, prior to sonication [14,18]. Ultrasound-assisted extraction (UAE) was carried out in a 1000 W ultrasound processor (Hielscher, UIP-1000 hdT, Teltow, Germany) for 2 min at 90 W/cm^2^ of sonication power [14], in a jacketed chamber, and cooled by a cold-water bath (Frigiterm circulation cryo-thermostat JP selecta, (Abrera, Barcelona, Spain) to maintain a blend temperature under 30 °C. Solid residue from all extracts was removed from liquid phase by centrifugation (high-speed laboratory centrifuge Retsch GmbH, ZM200, Haan, Germany) at 8500× *g* for 5 min and filtration through 0.45 µm membrane filters (Merck Millipore, Burlington, MA, USA); the supernatant was then stored at −20 °C until further use.

### 3.4. Extract Purification

An aliquot of WE was purified in a (2.5 cm × 47 cm) Bio-Rad column (Bio-Rad, Hercules, CA, USA) filled with Amberlite XAD16 resin. The working flux was set at 110 mL/h, using a peristaltic pump (Cole Parmer Masterflex, (Cole-Parmer Instrument Company LLC, Vernon Hills, IL, USA). Aqueous extracts (100 mL) were poured into the column and washed with 300 mL of distilled water; this fraction was mainly rich in uronic acids, and so it was discarded [10]. Then the column was flushed with 300 mL of ethanol–water 70% (*v*/*v*) to obtain a purified aqueous extract (PWE). Extracts were freeze-dried (FD) at −55 °C, 50 Pa, for 42 h and stored at RT, in darkness, until use.

### 3.5. Extract Oxidation

Three aliquots of PWE (10 g FD/L) were exposed to natural oxidation (PON), air oxidation (POA) pumping air with 80 L/h air flux and peroxide oxidation (POP) with a ratio of 1:1 (*v*/*v*) of hydrogen peroxide (36%). After 120 h at RT, all oxidized extracts were freeze-dried.

### 3.6. Extract Dialysis

Aliquots of PWE extract were dissolved in double-distilled water (10 g FD/L). Molecular-weight-cutoff dialysis was adapted from Tierney et al. [21] and was carried out by using 20, 10, 3.5 and 2 kDa Slide-A-lyzer G2 dialysis cassettes (Thermo Fisher Scientific, Waltham, MA, USA). Cassettes were clamped and immersed in a reservoir of double-distilled water and shaken (120 rpm) at 4 °C for 120 h. Both solutions, the high-molecular-weight (retentate) one and low-molecular-weight (dialysate) one, were freeze-dried.

### 3.7. Phytochemical Characterization

To determine proximate chemical content in all extracts, FD extracts were dissolved at 1 g FD/L in 20% (*v*/*v*) ethanol–water solution and filtered through 0.45 μm syringe filters. The total polyphenol content (TPC) was determined by using a calibration (R^2^ = 0.99) and expressed as g of phloroglucinol equivalents per liter (g PE/L). Extracts (100 µL) were mixed with 10% (*v*/*v*) Folin–Ciocâlteu reagent (500 µL) and 7.5 (*w*/*w*) sodium carbonate solution (400 µL) and were incubated at 40 °C for 15 min. The reaction was measured spectrophotometrically at 765 nm. The total carbohydrate (CHO) content of the extracts was analyzed by mixing 200 µL of extract with 5% (*v*/*v*) phenol (100 µL) and H_2_SO_4_ (1000 µL). Mixtures were incubated at 30 °C for 30 min, and the absorbance was measured spectrophotometrically at 485 nm. CHO values were evaluated by using a calibration with glucose as reference (R^2^ > 0.99) and expressed as g of glucose equivalents per liter (g GE/L). For the DPPH radical scavenging activity (RSA) analysis, extracts (20 μL) were mixed with 60 µM DPPH solution (980 μL). Measures were taken after an incubation time of 30 min, in complete darkness, at RT. The radical scavenging activity of samples was determined by using a calibration with Trolox (R^2^ > 0.99), measuring absorbance at 515 nm, and expressed as g of Trolox equivalents per liter (g TE/L). TPC, CHO and RSA spectroscopic determinations were measured with a Genesis 10S UV spectrophotometer (Thermo Fisher Scientific, USA) in triplicate, following a previously reported methodology by Gisbert et al. [14].

### 3.8. RP-HPLC–UV

Chromatographic separations were obtained by following a protocol proposed by Koivikko et al. [4], with minor modifications, and composed by a HPLC system (Jasco Applied Sciences, Hachioji City, Tokio 192-8537, Japan) equipped with a PU-980 pump, a quaternary gradient pump LG-1580-04, a UV-1575 detector and a degasser (Populaire DP4003). Data were obtained and processed with PowerChrom 2.5 software (eDaq Technologies, Denistone East, NSW 2112, Australia). Chromatographic separation was performed with a 250 × 8 mm Kromasil C18 semi-preparative column (Scharlab, Barcelona, Spain) (100 Å pore size and 5 µm particle size) at 35 °C. Mobile phase solvents were (A) water (0.1% (*v*/*v*) of formic acid) [18] and (B) methanol [10] filtered through 0.45 µm polyamide membrane filters (Sartorius, Göttingen, Germany) and degassed in an ultrasonic bath Elmasonic S40 (Elma Schmidbauer GmbH, Singen Germany). The elution profile was set at 0–30 min (100% A), 31–70 min (100% B) and 71–100 (100% A). The mobile phase profile was set as solvent change to ensure the separation of molecule families. Samples (10 g FD/L) were diluted in water–ethanol 20% (*v*/*v*) and filtered through 0.22 µm syringe filters (Macherey-Nagel GmbH & Co. KG, Dueren, Germany) prior to the injections. The injection volume was 20 µL, the detection wavelength was 266 nm [41] and the solvent flow rate was set at 0.4 mL/min. To ensure the reproducibility of the assays, they were carried out in duplicate. Standard molecules of phloroglucinol and resorcinol were injected to confirm the presence of phlorotannins. Glucose and glucuronic acid standards were injected to check for possible interference of carbohydrates’ isoform, and bovine serum albumin (BSA) was used as protein control.

### 3.9. FTIR

Fourier-transformed infrared (FTIR) spectrophotometry was employed to determine chemical changes among the extracts and their fractions [32]. FTIR spectra were recorded in a Bruker FT-MIR model Vertex 70 V spectrometer. Wave number range was set in the range of 800 to 4000 cm^−1^. Samples were blended with KBr and compressed into disks. FTIR spectra analysis was carried out by using Omnic 7.1 software (Thermo Fisher Scientific, USA).

### 3.10. ^1^H-NMR

Proton nuclear magnetic resonance spectroscopy (^1^H-NMR) spectra were obtained with a Bruker NEO 750 spectrometer that was operated with a 17.61 T (750 MHz resonance ^1^H) magnetic-field strength. Extracts were dissolved in DMSO-d_6_, and ^1^H-RMN spectra data treatment was carried out with MestreNova software (Mestrelab Research, A Coruña, Spain). The spectra were analyzed by following Breton et al.’s [9] and Audibert et al.’s [27] studies.

### 3.11. MALDI–TOF-MS

The mass detectors currently used chromatographic systems (liquid and mass), have a detection limit of approximately 2000 Da and become a limiting factor when working with phlorotannins, which are known to be larger. Then, for the analysis of oligomers, above 500 Da in the extracts was carried out with a matrix-assisted laser desorption/ionization–time-of-flight mass spectrometry (MALDI–TOF-MS). MALDI–TOF-MS spectra were recorded for samples PWE, PD20 and PD2 to assess the range of mass fragmentation. The equipment used was Bruker Ultraflex III TOF/TOF equipped with a N_2_ laser of 337 nm, 1 µm of laser beam diameter and operated in positive mode. MALDI–TOF-MS spectra data treatment was carried out with data-analysis software (Bruker, Billerica, MA, USA). MALDI–TOF-MS analysis was performed by following a protocol that was previously proposed [10].

### 3.12. Statistical Analysis

Statistical analyses were carried out by using IBM SPSS statistics 27 (SPSS Inc., Chicago, IL, USA) software. A one-way analysis of variance (ANOVA) was assessed based on a confidence interval of 95% (*p* < 0.05), using a Duncan test. The experimental results were treated and plotted on Microsoft Excel (Microsoft Corporation, Redmond, WA, USA). All experimental results were expressed as mean ± standard deviation of triplicate experiments.

## 4. Conclusions

Bioactive (TPC, CHOs and RSA), chromatographic (RP-HPLC) and spectroscopic (FTIR, ^1^H-NMR and MALDI-TOF-MS) results confirmed that aqueous mixture of acetone (AWE) was a better extractant of phlorotannins than water (WE), but it renders an extract with a significant amount of carbohydrates. Purification of extract (PWE) reduced the carbohydrate content (i.e., removing high-molecular-size alginate oligomers), leaving a similar polyphenol content but with higher antioxidant activities than WE and comparable to AWE. 

Radical scavenging activity (RSA) was maintained after oxidation with air and only hydrogen peroxide oxidation (POP) was able to achieve a significant oxidation of phlorotannins in PWE extract. The analysis of dialyzed extracts showed that phlorotannins with a molecular size in the range of 10–20 kDa were main bioactive phlorotannins, with TPC and RSA values of 49.7 and 53.6%, respectively. Hence, the lowest-molecular-size extract (PD2) showed low bioactivity, which was in the range that of oxidized POP. Future work will involve a more in-depth profiling analysis of the phlorotannins to assist in the complete characterization of their structures partially subjected to controlled oxidation.

## Figures and Tables

**Table 1:** Phloroglucinol units of dialyzed PD20 and PD2 extracts in comparison with extracts from brown seaweeds reported by bibliography.

Literature Reference	*Seaweed* spp.	Main DP Range
This study (PD2)	*A. nodosum*	4–17
Steevensz et al. [8]	*A. nodosum*	4–39
Allwood et al. [10]	*A. nodosum*	11–18
Kadam et al. [17]	*A. nodosum*	4–12
Tierney et al. [21]	*A. nodosum*	6–11
Sardari et al. [36]	*A. nodosum*	3–6
Catarino et al. [18]	*Fucus vesiculosus*	5–10
Bogolitsyn et al. [31]	*Fucus vesiculosus*	3–8
Lopes et al. [37]	*Fucus vesiculosus*	3–6
Hefferman et al. [38]	*Fucus vesiculosus*	3–16
Vissers et al. [34]	*Laminaria digitata*	3–27

**Table 2:** List of phlorotannins identified in (Figure 7) and water loss n[H_2_O], comparing data with Steevensz et al. [8].

Formula	Steevensz et al. [8]	[H_2_O] Loss	This Study (m/z)	DP
C_30_H_22_O_15_	621	5	529 ^a^	4
	621	2	586 ^b^	4
C_36_H_26_O_18_	745	3	691 ^a^	5
C_84_H_58_O_42_	868	1	853 ^a^	5
C_90_H_62_O_45_	930	0	927 ^c^	6
C_150_H_102_O_75_	1033	1	1015 ^a^	6
C_114_H_78_O_57_	1178	0	1177 ^a^	7
C_264_H_178_O_132_	1364	1	1339 ^a^	8
C_294_H_198_O_147_	1519	1	1501 ^a^	9

Superscript letters were used to identify different phlorotannins families within the extract.

## Data Availability

Data are available in the present paper and as supplementary material.

## Supplementary Materials

The following are available online at https://www.mdpi.com/article/10.3390/md20110706/s1, Figure S1: Detailed full-scale RP-HPLC–UV profiles (266 nm) of (A) standard compounds (phloroglucinol, resorcinol, glucose, glucuronic acid and BSA); (B) crude and purified (AWE, WE and PWE), (C) oxidized, (PON, POA and POP) and dialyzed (PD20, PD10, PD3 and PD2) extracts.
