# Supplementary figures and images for: Influence of Oxidation and Dialysis of Phlorotannins on Bioactivity and Composition of Ultrasound-Assisted Extracts from Ascophyllum nodosum
Source: Mar Drugs. 2022 Nov 11;20(11):706. doi: 10.3390/md20110706 (PMC9695499; doi:10.3390/md20110706)

# Phloroglucinol

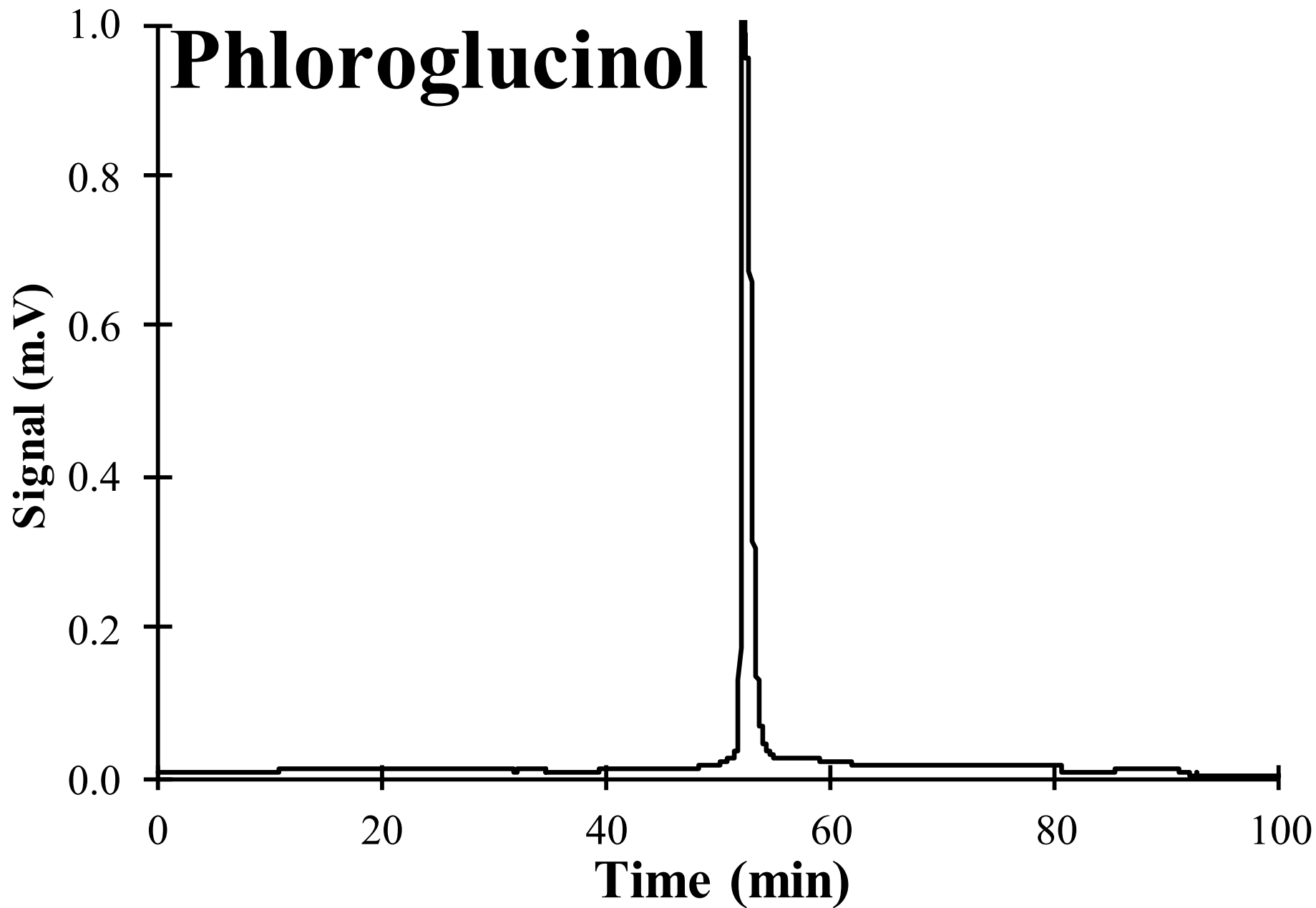

# Resorcinol

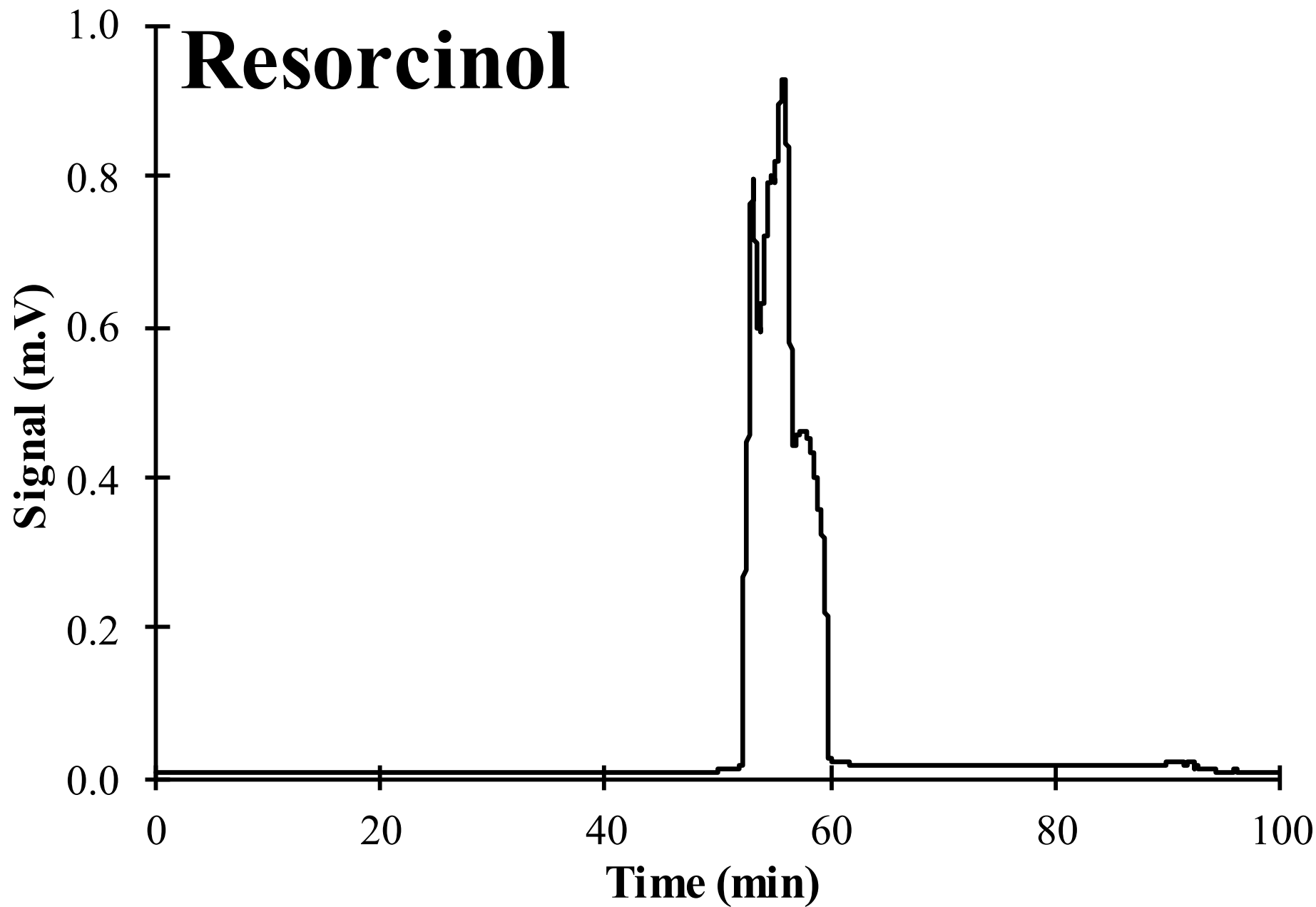

# Glucose

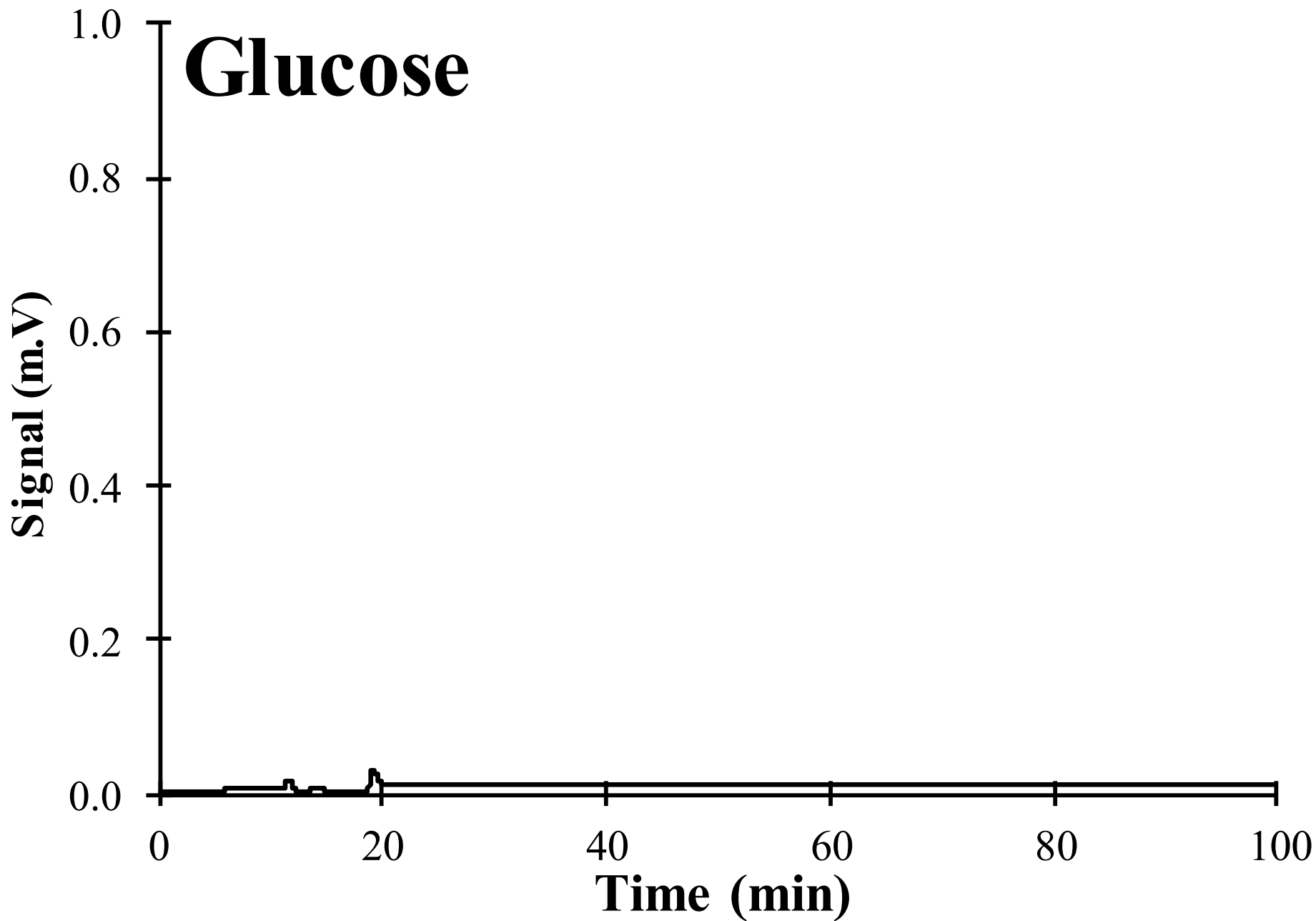

# Glucuronic acid

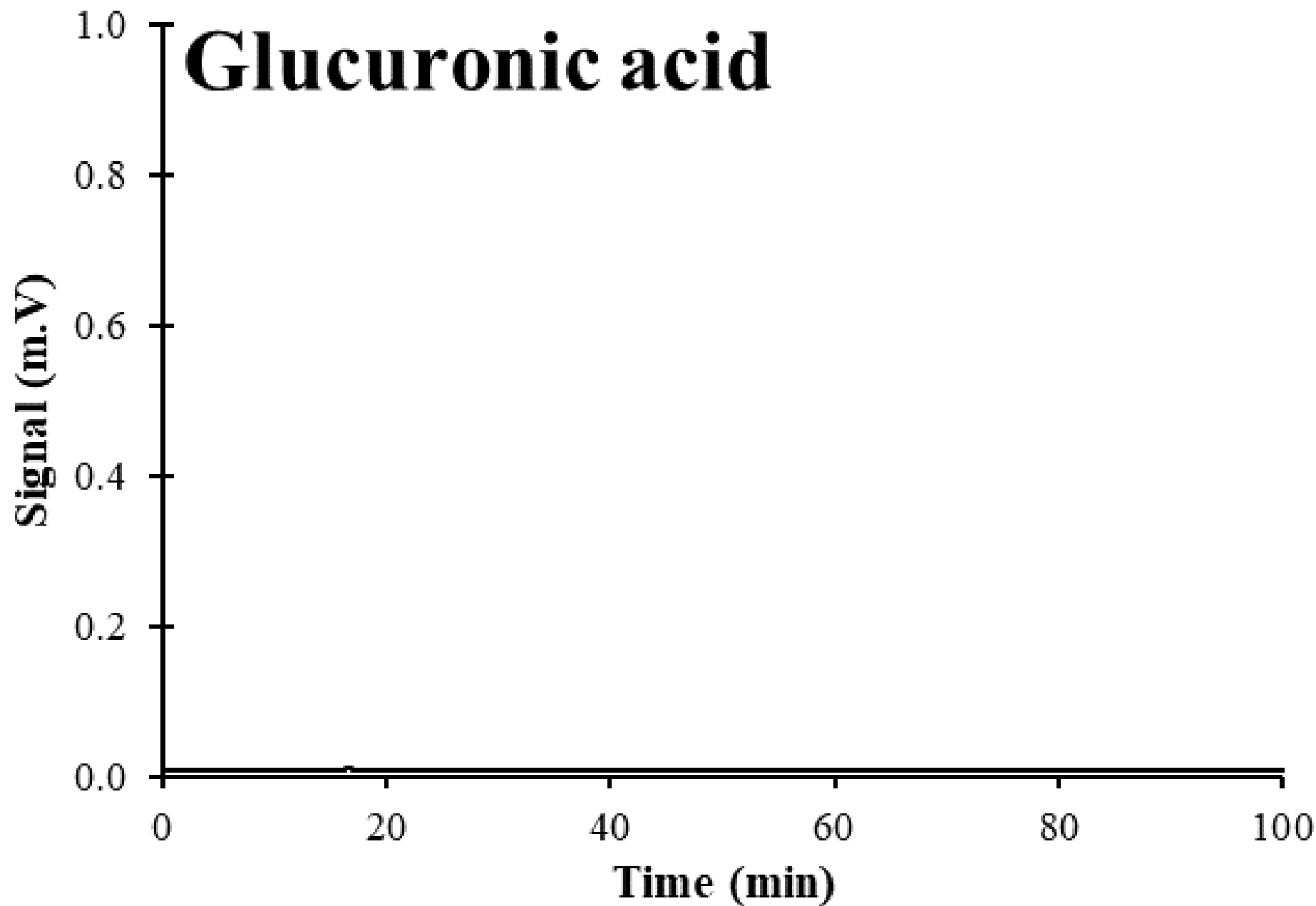

**BSA**

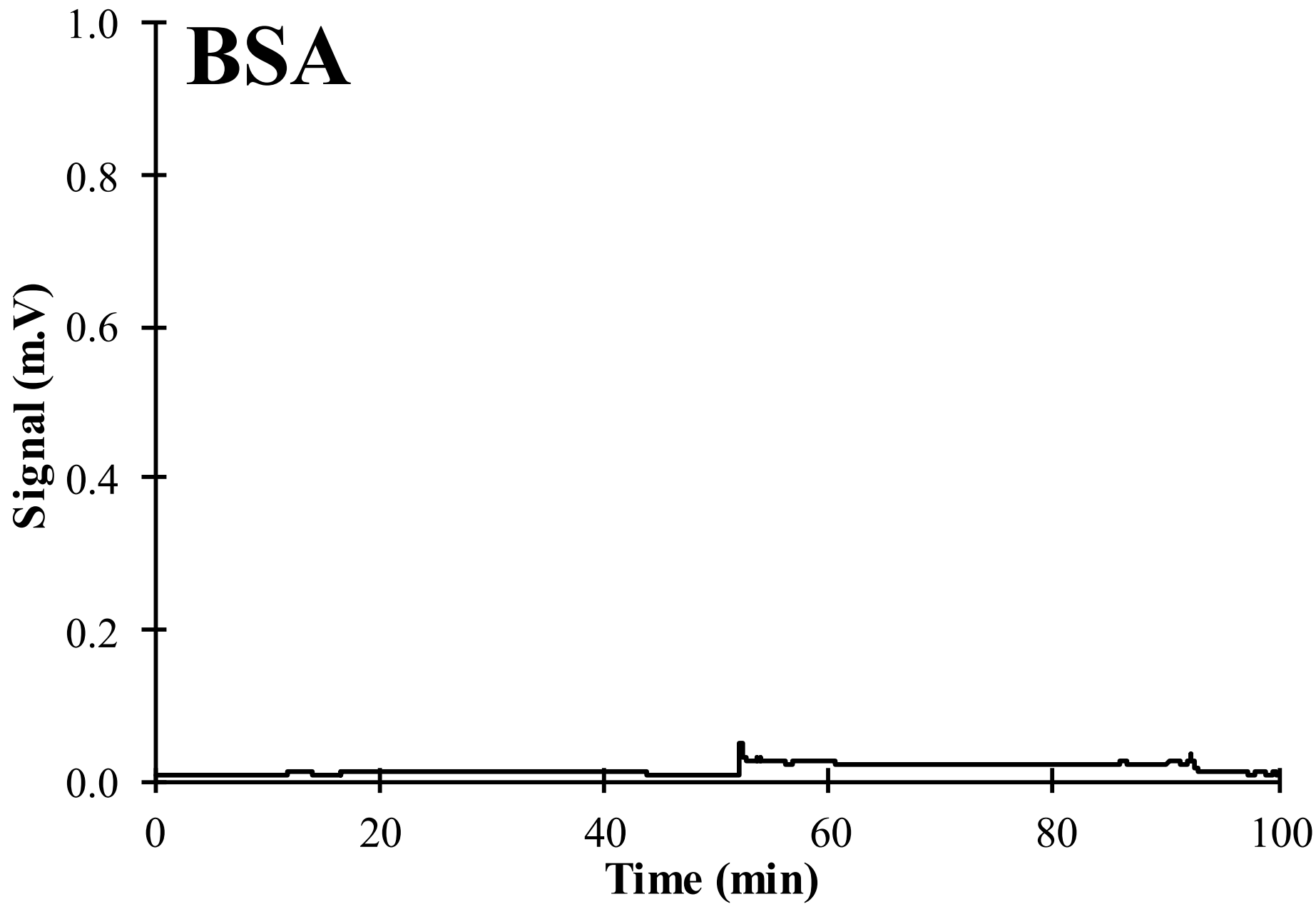

**AWE**

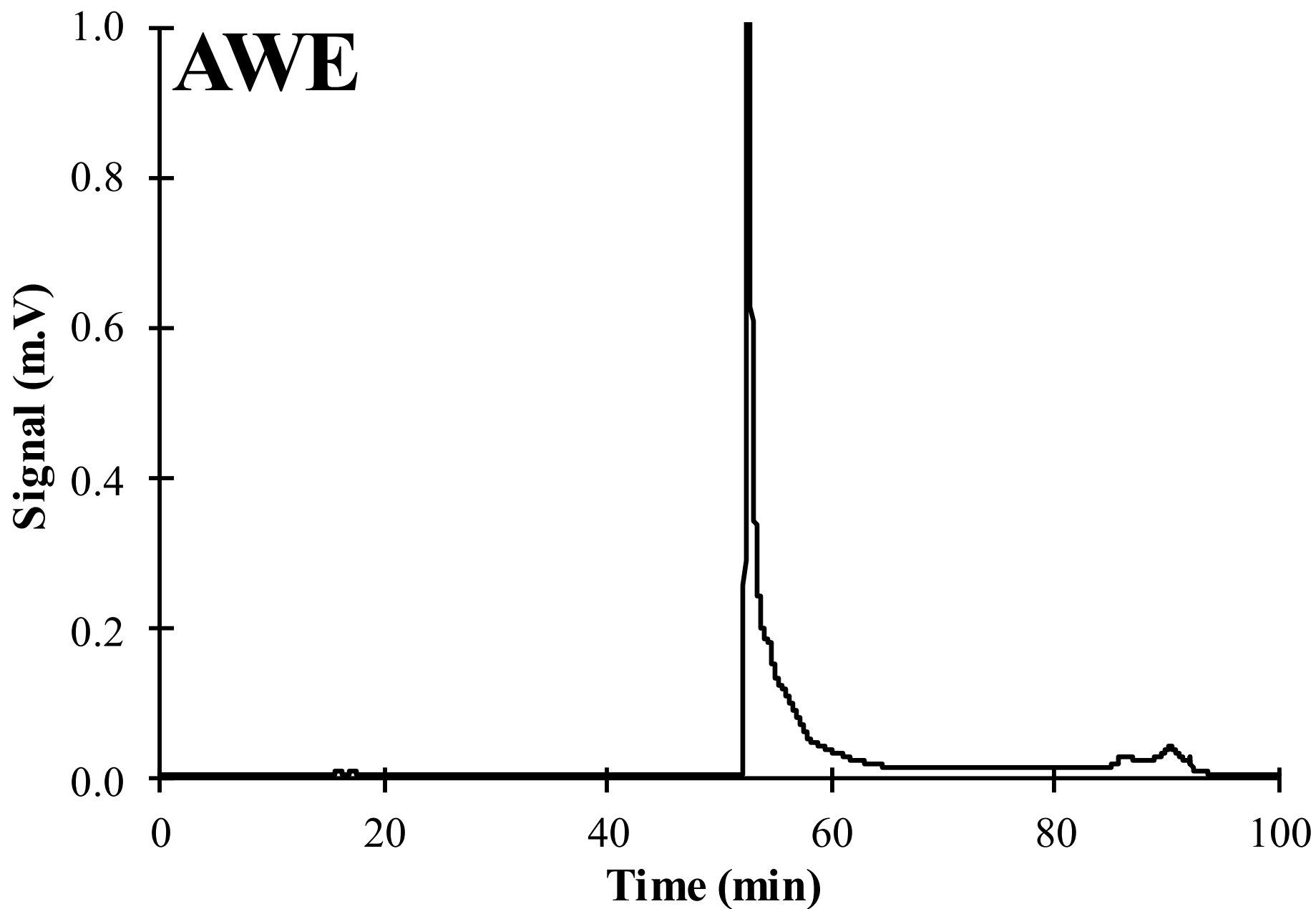

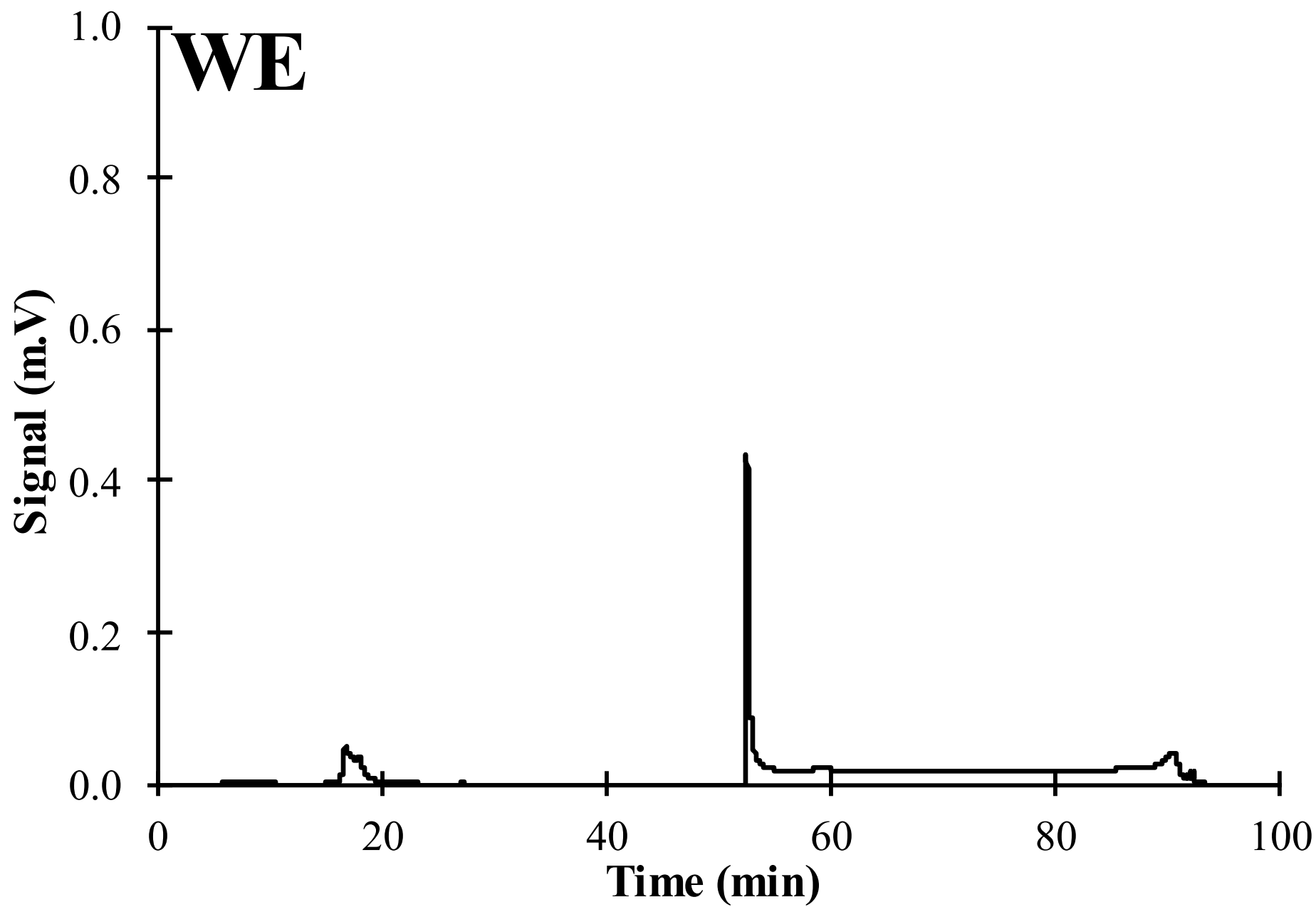

**PWE**

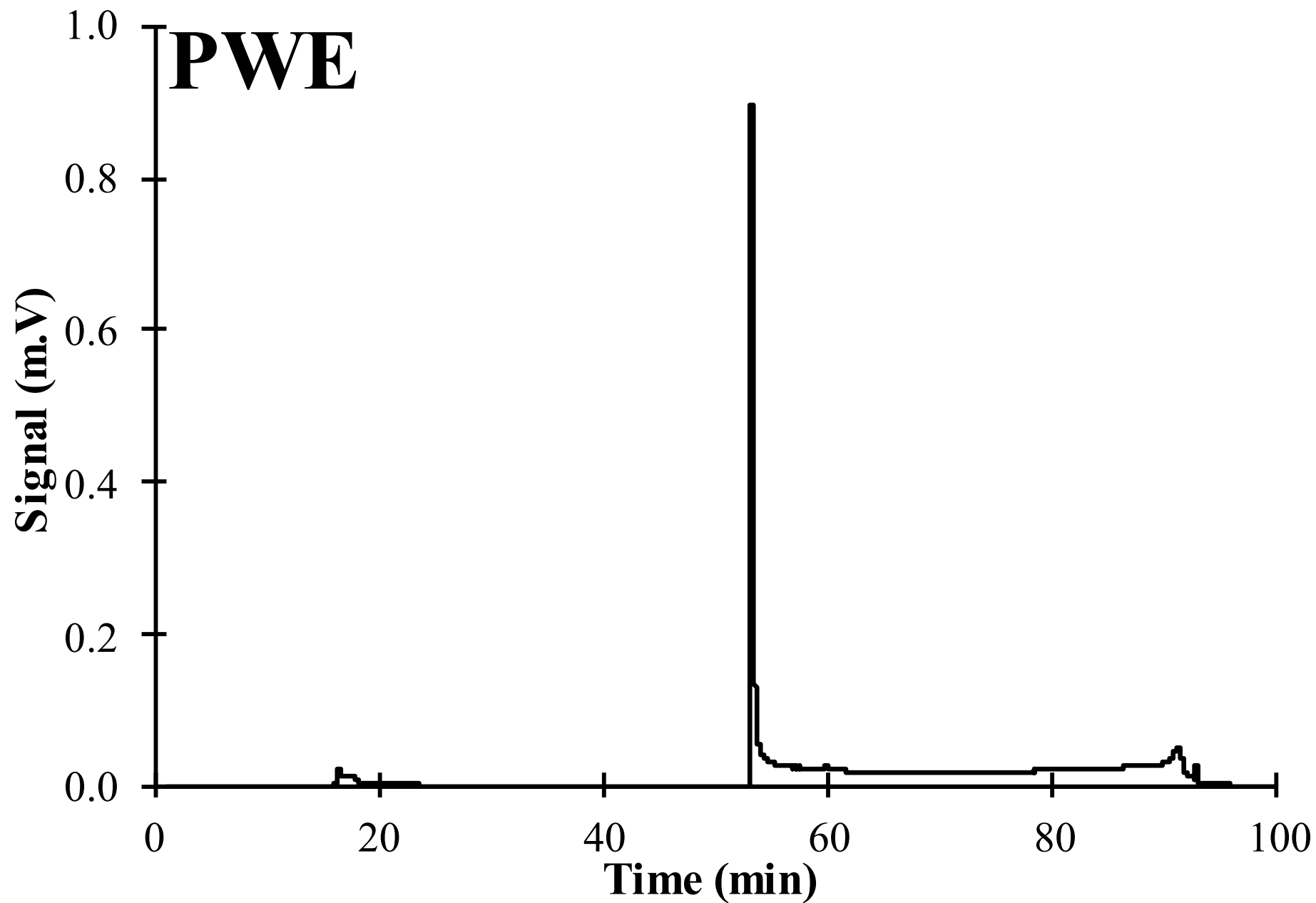

**PON**

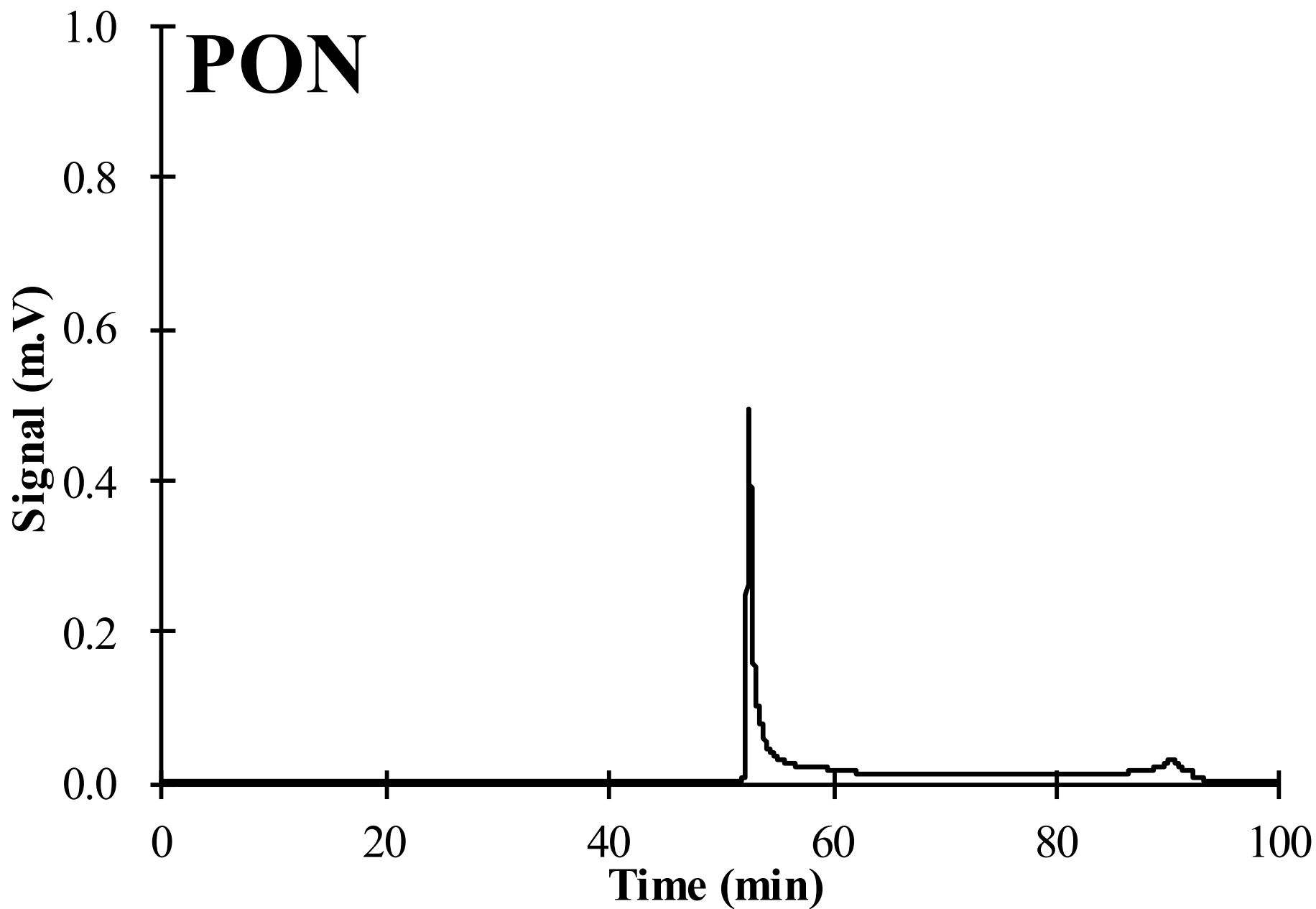

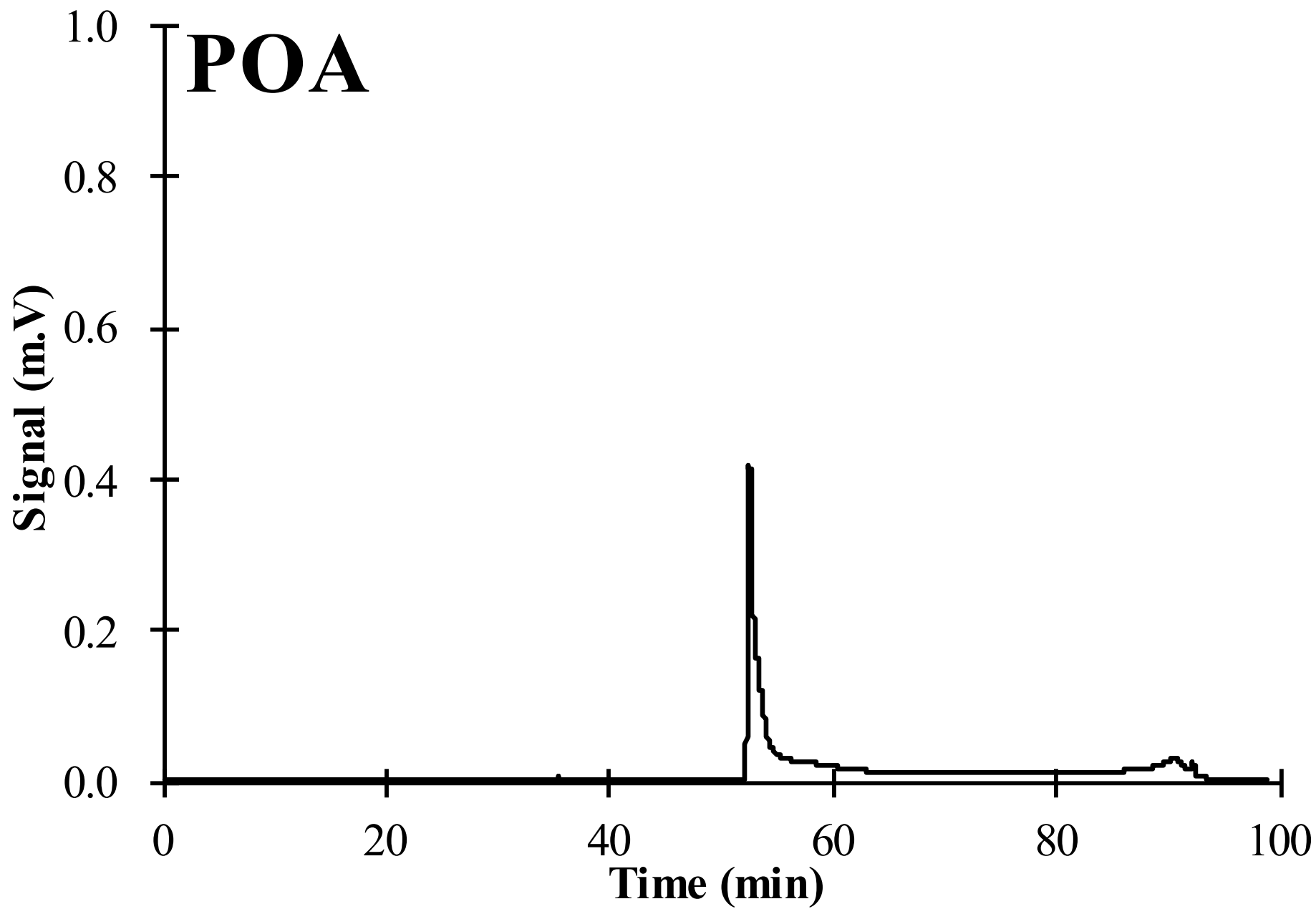

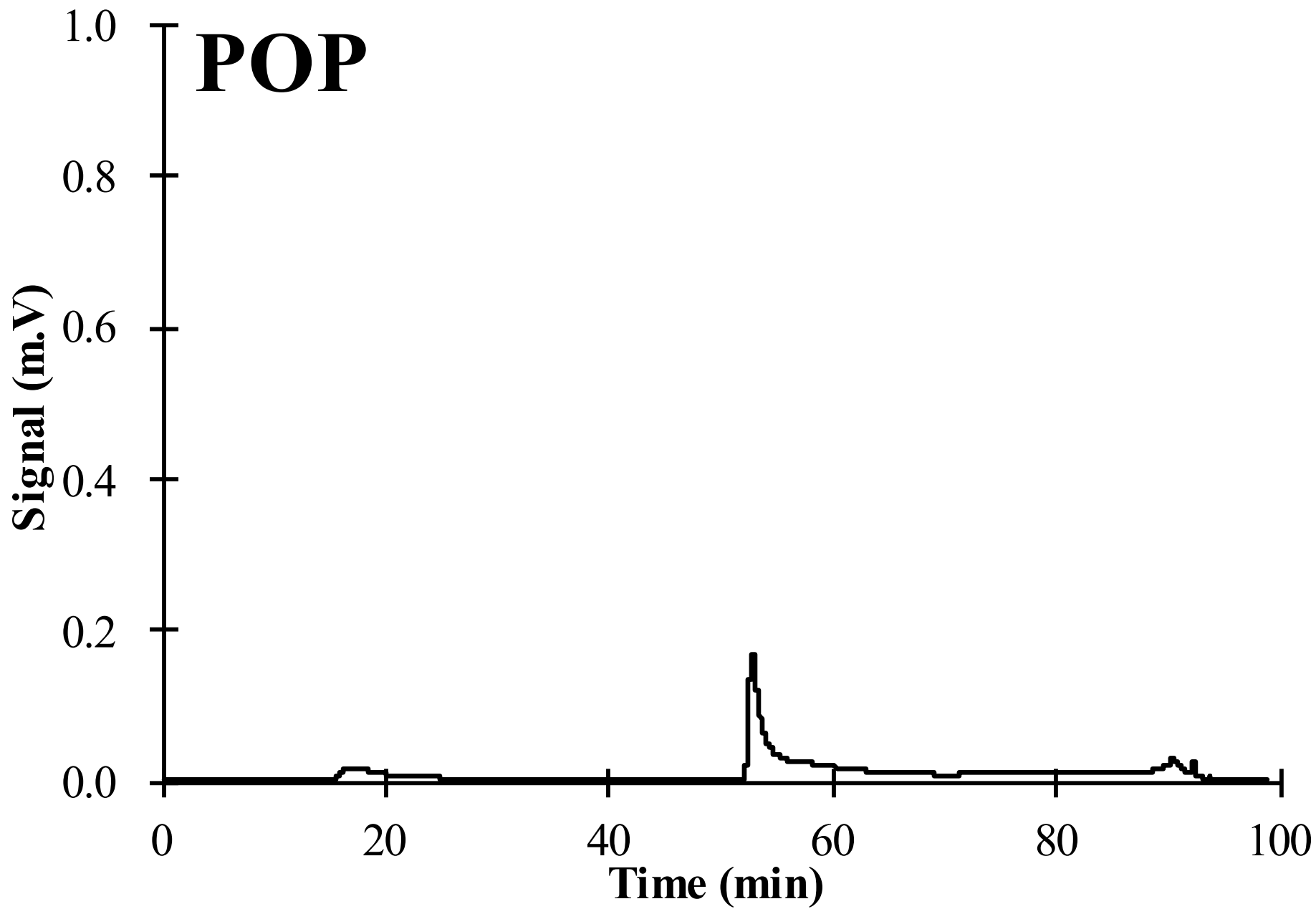

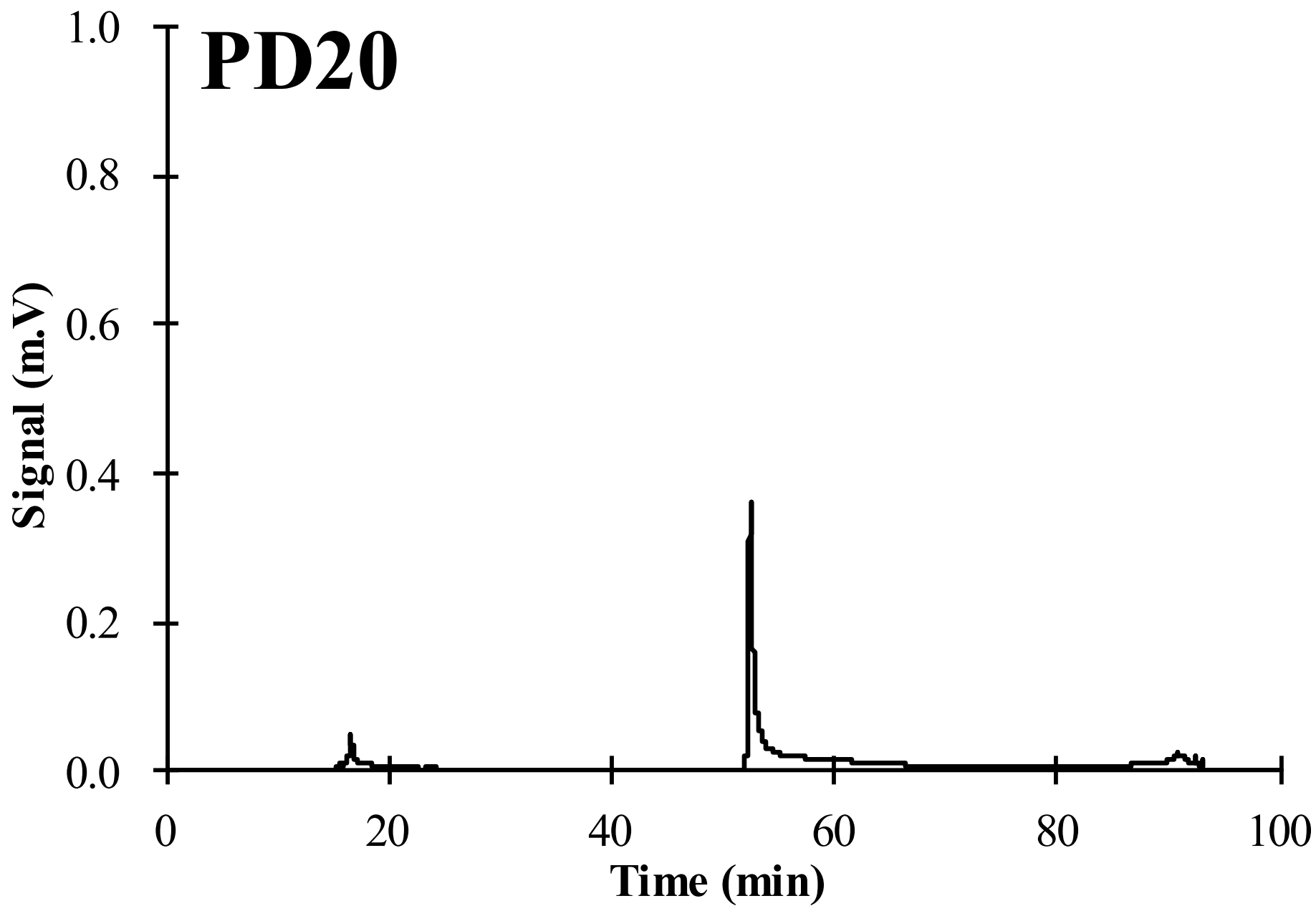

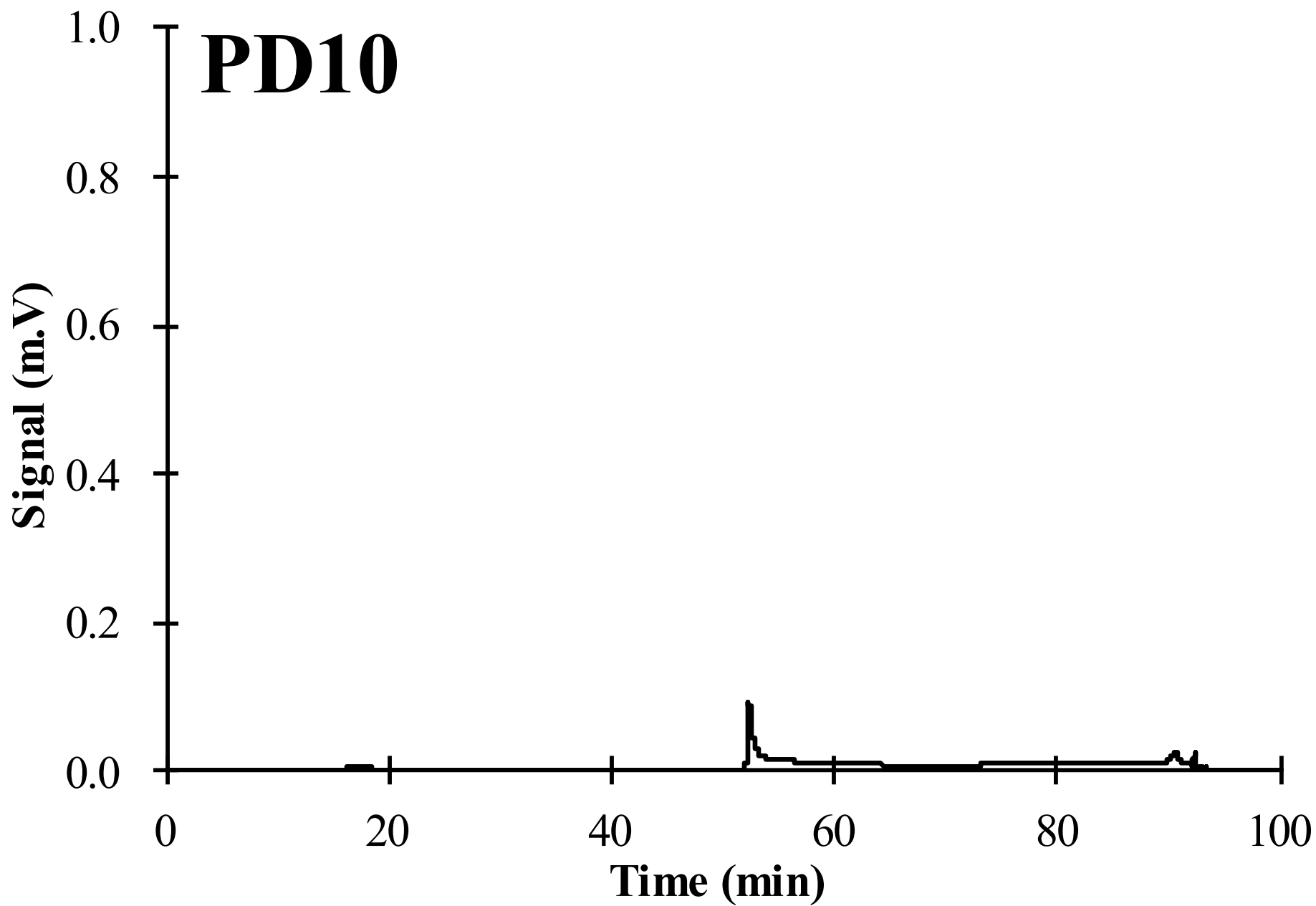

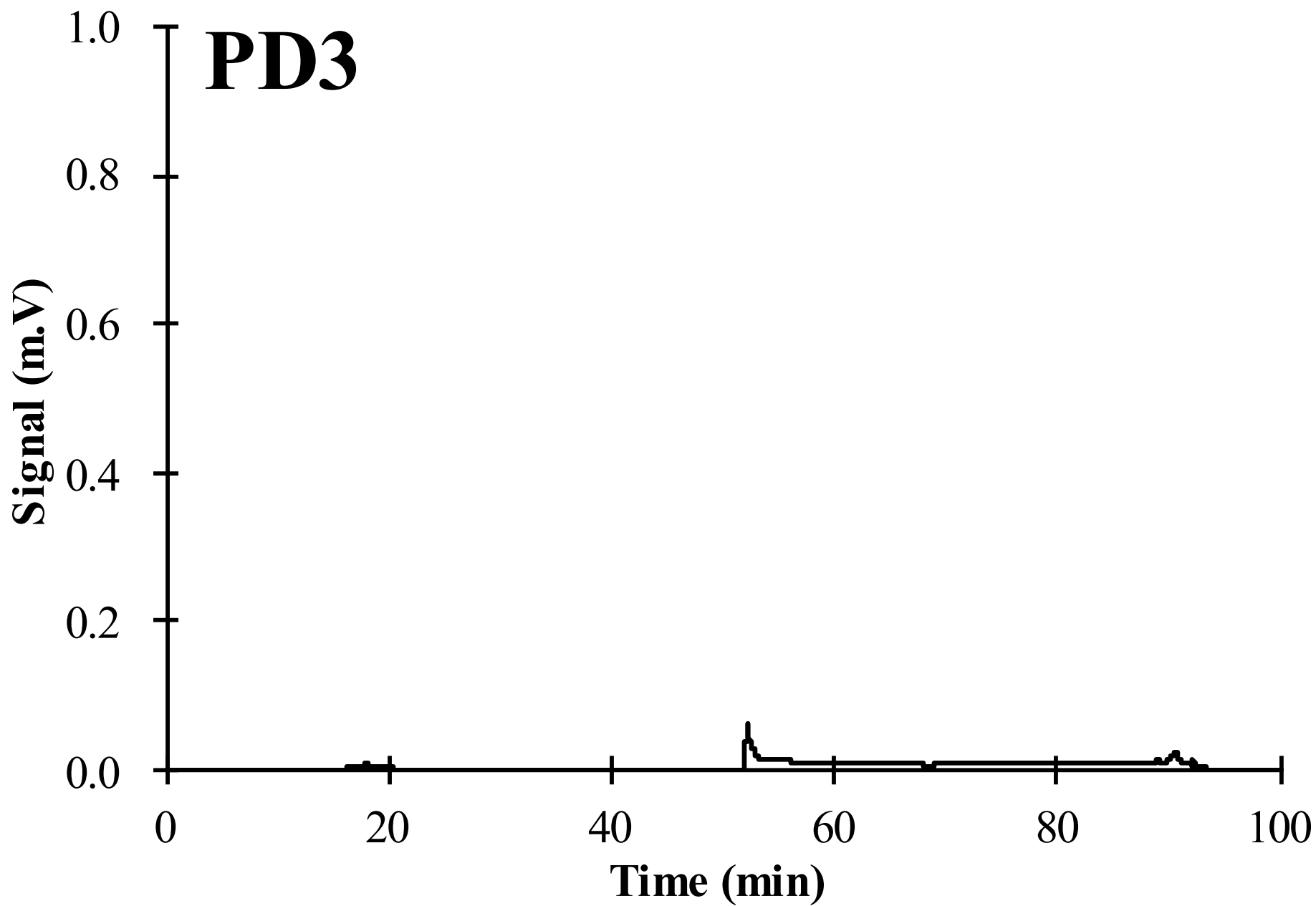

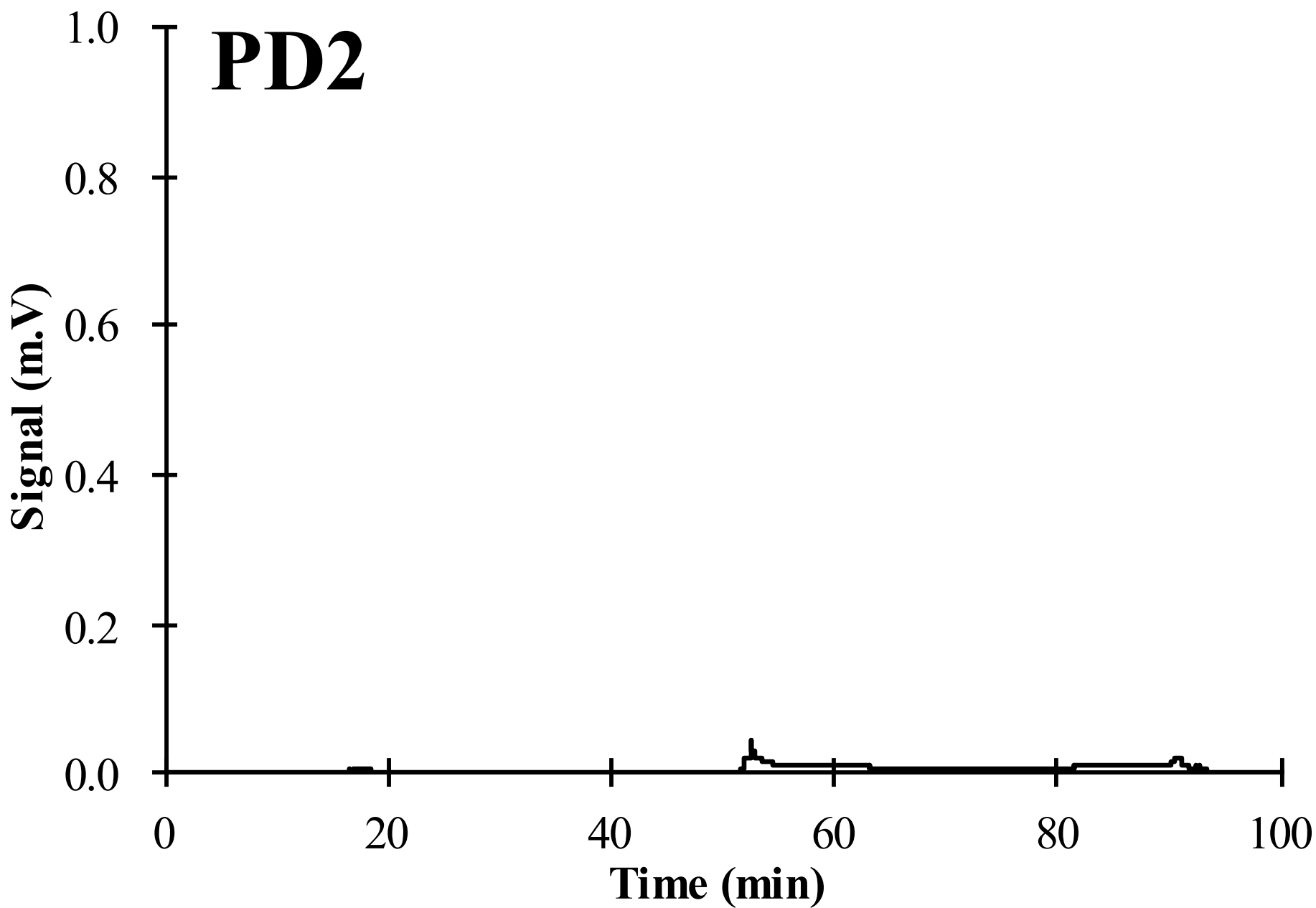

Supplement: Supplementary file 1 [file marinedrugs-20-00706-s001.zip › marinedrugs-1991929-supplementary-Figure S1.pdf]
